# Transfer of a human gene variant associated with exceptional longevity improves cardiac function in obese type 2 diabetic mice through induction of the SDF‐1/CXCR4 signalling pathway

**DOI:** 10.1002/ejhf.1840

**Published:** 2020-05-08

**Authors:** Zexu Dang, Elisa Avolio, Anita C. Thomas, Ashton Faulkner, Antonio P. Beltrami, Celeste Cervellin, Albino Carrizzo, Anna Maciag, Yue Gu, Elena Ciaglia, Nicoletta Finato, Antonio Damato, Gaia Spinetti, Aishah Alenzi, Stephen J. Paisey, Carmine Vecchione, Annibale A. Puca, Paolo Madeddu

**Affiliations:** ^1^ Translational Health Sciences, Bristol Medical School University of Bristol Bristol UK; ^2^ Department of Medicine University of Udine Udine Italy; ^3^ Vascular Pathophysiology Unit, IRCCS Neuromed Pozzilli Italy; ^4^ Cardiovascular Department IRCCS Multimedica Milan Italy; ^5^ Department of Medicine, Surgery and Dentistry, “Scuola Medica Salernitana” University of Salerno Baronissi (SA) Italy; ^6^ PETIC, School of Medicine University of Cardiff Cardiff UK

**Keywords:** Cardiomyopathy, Diabetes, Longevity, Gene therapy, BPIFB4

## Abstract

**Aims:**

Homozygosity for a four‐missense single‐nucleotide polymorphism haplotype of the human *BPIFB4* gene is enriched in long‐living individuals. Delivery of this longevity‐associated variant (LAV) improved revascularisation and reduced endothelial dysfunction and atherosclerosis in mice through a mechanism involving the stromal cell‐derived factor‐1 (SDF‐1). Here, we investigated if delivery of the *LAV‐BPIFB4* gene may attenuate the progression of diabetic cardiomyopathy.

**Methods and results:**

Compared with age‐matched lean controls, diabetic db/db mice showed altered echocardiographic indices of diastolic and systolic function and histological evidence of microvascular rarefaction, lipid accumulation, and fibrosis in the myocardium. All these alterations, as well as endothelial dysfunction, were prevented by systemic *LAV‐BPIFB4* gene therapy using an adeno‐associated viral vector serotype 9 (AAV9)*.* In contrast, AAV9 wild‐type‐*BPIFB4* exerted no benefit. Interestingly, *LAV‐BPIFB4*‐treated mice showed increased SDF‐1 levels in peripheral blood and myocardium and up‐regulation of the cardiac myosin heavy chain isoform alpha, a contractile protein that was reduced in diabetic hearts. SDF‐1 up‐regulation was instrumental to *LAV‐BPIFB4‐*induced benefit as both haemodynamic and structural improvements were inhibited by an orally active antagonist of the SDF‐1 CXCR4 receptor.

**Conclusions:**

In mice with type‐2 diabetes, *LAV‐BPIFB4* gene therapy promotes an advantageous remodelling of the heart, allowing it to better withstand diabetes‐induced stress. These results support the viability of transferring healthy characteristics of longevity to attenuate diabetic cardiac disease.

## Introduction

Diabetic cardiomyopathy has a complex pathogenic basis and is therefore difficult to treat by targeting a single candidate mechanism.^1^, ^2^ The genetics of healthy longevity may inspire novel treatments for this condition. Long‐living individuals are protected from the consequences of age‐related pathologies. Moreover, their offspring show a lower incidence of cardiovascular disease, suggesting the healthy phenotype can be transmitted vertically through generations.^3^ We postulate that horizontal transfer of genes associated with healthy longevity may be a novel way to fight cardiovascular complications in people with diabetes.

Genome‐wide association studies have led to the discovery of polymorphic gene variants linked to exceptional longevity.^4^ One such study revealed that a four‐missense single‐nucleotide polymorphism variant of the bactericidal/permeability‐increasing fold‐containing family B member 4 (*BPIFB4*) associates with extraordinarily prolonged life‐span in three different geographic areas.^5^ Longevity‐associated variant (*LAV*)*‐BPIFB4* has an allele frequency of 29.5%, while the wild‐type isoform (*WT‐BPIFB4)* allele frequency is around 66% in the Caucasian population. The prevalence of LAV homozygosity is 14% in centenarians and 10% in controls.^6^

BPIFB4 is a secreted protein, its levels are increased in serum of long‐living individuals, and high BPIFB4 levels classify their health status.^7^ Likewise, homozygous *LAV* carriers have higher circulating BPIFB4 levels and increased phosphorylated endothelial nitric oxide synthase (eNOS) in circulating mononuclear cells.^6^, ^7^ Thus, enhanced qualities and increased circulating quantities may account for the benefit of carrying the *LAV* rather than the *WT* gene.

Systemic delivery of *WT‐BPIFB4* or *LAV‐BPIFB4* using an adeno‐associated viral vector (AAV) induced similar over‐expression of the human protein in most tissues of transfected mice, except for an increase of the LAV protein in peripheral blood and circulating monocytes. Importantly, *LAV‐BPIFB4*, but not *WT‐BPIFB4*, protected from hypertension, ischaemia and atherosclerosis.^5^, ^8^ Investigation of mechanisms behind this remarkable superiority indicated that *LAV‐BPIFB4* is (i) more efficiently phosphorylated/activated by protein kinase R‐like endoplasmic reticulum kinase and protein kinase C alpha, (ii) activates a feedforward mechanism that involves increased calcium mobilisation, and (iii) has greater capacity in binding 14–3‐3, recruiting heat shock protein 90, and eventually activating eNOS.^9^ Additionally, the anti‐atherosclerosis action of *LAV‐BPIFB4* gene therapy was attributed to a mechanism involving the stromal cell‐derived factor‐1 (SDF‐1) and related CXCR4 receptor.^8^

The present study investigates the potential of *LAV‐BPIFB4* gene therapy and participation of SDF‐1/CXCR4 axis in halting the progression of diabetic cardiomyopathy.

## Methods

### Adeno‐associated viral vector production

The vector was produced as described previously.^5^ For each viral preparation, physical titres (GC/mL) were determined through dot‐blot analysis and polymerase chain reaction quantification using TaqMan20 (Applied Biosystems, Carlsbad, CA, USA).

### Studies on human cardiac tissue

The study was approved by the Ethics Committee of the University Hospital of Udine, Italy (number 47831) in accordance with the Declaration of Helsinki.

BPIFB4 expression was assessed in hearts explanted from patients undergoing transplantation for ischaemic heart failure. Hearts of patients that died for causes other than cardiovascular disease or donated hearts not employed for transplantation were used as controls (online supplementary *Table S1*). Formalin‐fixed and paraffin‐embedded tissues were cut into 5 μm thick sections and stained for BPIFB4, CD34 and α‐sarcomeric actin (online supplementary *Table S2*). Images were acquired with a Leica DMI6000B microscope (Leica Microsystems, Wetzlar, Germany) and processed using ImageJ software (https://imagej.net/ImageJ).

### Protocols in mice

Experimental procedures were approved by the British Home Office (PPL numbers 30/2811 and 30/3373), the University of Bristol, the IRCCS Neuromed Animal Care Review Board, and the Istituto Superiore di Sanità, Rome (1163/2015‐ PR), and were compliant with the EU Directive 2010/63/EU and principles stated in the Guide for the Care and Use of Laboratory Animals (Institute of Laboratory Animal Resources, 1996). Mice were housed in groups of 3–6 animals or individually (as required by the experimental procedure) in an enriched environment within a bio‐secure unit under a 12 h light/dark cycle, fed with EURodent Diet (5LF5, LabDiet, Durham, UK) and given drinking water ad libitum.

#### *Ex vivo* transfection of mouse vessels

Second‐order branches of the mesenteric artery were transfected with plasmid vector as described.^5^, ^8^ Vessels were placed in a Mulvany pressure system filled with Krebs solution supplemented with 20 μg of the pRK5 vector encoding either empty, *WT*‐ or *LAV‐BPIFB4* gene. Endothelium‐dependent and independent relaxation was assessed by measuring the dilatory responses to cumulative acetylcholine concentrations (from 10^−9^ M to 10^−5^ M) in vessels pre‐contracted with U46619 at a dose necessary to obtain a similar level of pre‐contraction (80% of initial KCl‐evoked contraction) and reported as a percentage of diameter change. Responses were tested before and after transfection, in presence or absence of high glucose concentration (25 mM for 1 h), and pre‐treatment with the SDF‐1 antagonist AMD3100 (1 μM) or vehicle for 30 min.

#### *In vivo* studies

- *Study 1: Effect of LAV‐BPIFB4 gene transfer on cardiac function of non‐diabetic mice*. Nine‐week‐old male C57BL/6J mice (Charles River, Margate, Kent, UK) under isoflurane anaesthesia received 100 μL of 1 × 10^12^ GC/mL *AAV9‐LAV‐BPIFB4* or vehicle (PBS) (*n* = 6 per group) via the tail vein. Four weeks later, animals underwent echocardiography under general anaesthesia, followed by termination for tissue harvesting.
- *Study 2: Comparison of the effect of a single intravenous injection of AAV9‐LAV‐ or WT‐BPIFB4 in type 2 diabetic mice*. One week after baseline echocardiography, 9‐week‐old male C57BLKS/J‐Leprdb/Leprdb/Dock7+ [db/db] mice (Envigo, Bicester, Oxfordshire, UK) were intravenously injected, under isoflurane anaesthesia, with either *AAV9‐LAV‐BPIFB4*, *AAV9‐WT‐BPIFB4* (100 μL at 1 × 10^12^ GC/mL) or vehicle (PBS). Each treatment group consisted of 8 mice. Age‐matched lean non‐diabetic mice [C57BLKS/J‐Leprdb/LeprWT Dock7+ (wt/db), Envigo] were used as controls (*n* = 8). Four weeks later, mice underwent a final echocardiography followed by blood and tissue collection for molecular and histological analyses. Using the same randomisation protocol, mice underwent a cardiac positron emission tomography/computed tomography (PET/CT) scan under terminal anaesthesia to assess the myocardial uptake of 2‐deoxy‐2‐[fluorine‐18]fluoro‐D‐glucose (18F‐FDG).

An additional cohort of singly‐housed 28 diabetic mice was randomised to receive *AAV9‐LAV‐BPIFB4* or *AAV9‐GFP* (100 μL at 1 × 10^12^ GC/mL) control virus through the tail vein in association with 2 mg/kg/day AMD‐070 (Generon, UK), a specific CXCR4 antagonist, or DMSO (vehicle) orally in sugar‐free jelly. Mice were conditioned to eat the jelly for 1 week before entering the study; the jelly was given daily and intake was monitored closely throughout the study. Four weeks later, mice were sacrificed after a terminal echocardiography.

- *Study 3: Effect of AAV9‐LAV‐BPIFB4 gene therapy on systemic blood pressure and endothelial function*. Nine‐week‐old male db/db and wt/db mice (Envigo) were randomly attributed to receive *AAV9‐LAV‐BPIFB4* (100 μL at 1 × 10^12^ GC/mL) or *AAV9‐GFP* control virus (100 μL at 1 × 10^12^ GC/mL) (*n* = 5 per group) through the tail vein. Blood pressure was monitored using tail‐cuff plethysmography.^10^ Four weeks post‐treatment, mesenteric arteries were excised for assessment of vascular reactivity and molecular analyses.^11^ Vasorelaxation was evaluated using increasing dosages of acetylcholine and nitroglycerine (10^−9^ to 10^−6^ M) (Sigma‐Aldrich).

### Haemodynamic measurements and imaging

#### Echocardiography

Dimensional and functional parameters were measured by two independent investigators (Z.D. and A.C.T.) using a Vevo‐3100 echocardiography system (Fujifilm VisualSonics Inc, Toronto, Canada) and MX400 or MX550D transducers according to the animal size.^12^, ^13^ The study was performed with mice under isoflurane anaesthesia (2.5% for induction, followed by 0.5–1.2% as appropriate to maintain heart rate close to 450 bpm.

#### Positron emission tomography/computed tomography imaging

The study was conducted at PETIC, Cardiff University, UK, using a Mediso nanoScan 122S small bore PET/CT imaging system (Mediso Medical Imaging Systems, Budapest, Hungary). The protocol was standardised to ensure optimal and consistent biodistribution of 18F‐FDG. Briefly, mice had food withdrawn and were warmed at 37°C 1 h before scanning, which was done under isoflurane‐anaesthesia (∼2% in 1 L/min oxygen). An intraperitoneal injection of 100–150 μL of Iohexol CT contrast agent (647 mg/mL) (Omnipaque 300, GE Healthcare Inc., Marlborough, MA, USA) was followed by a tail vein injection of 32 ± 8 MBq 18F‐FDG. A 20‐min cardiac gated PET scan was acquired followed by a 2.5 min whole‐body CT scan. Cardiac 18F‐FDG uptake was expressed as % injected dose.

#### Biochemical measurements

Peripheral blood levels of C‐peptide and insulin were assessed using a Mouse Milliplex kit (MMHMAG‐44 K, Millipore, Burlington, MA, USA), atrial natriuretic peptide (ANP) was determined using EIA (RAB00385, Sigma‐Aldrich), and SDF‐1 was measured using an ELISA kit (CXCL12/SDF‐1 Quantikine ELISA Kit, DY350‐05, R&D Systems). BPIFB4 protein levels were determined using an ELISA kit (CSB‐EL003694HU, Cusabio Technology, Houston, TX, USA). According to the vendor, the ELISA has an excellent specificity for the human protein, but cross‐reaction with the mouse protein cannot be excluded.

### Histological analyses of mouse hearts

Sections were stained with specific antibodies employing the procedures listed in online supplementary *Table S2* and analysed using optical or fluorescence microscopy. Dimensional measures of nucleated cardiomyocytes were acquired using morphometry. Cardiomyocyte length and transversal diameter were analysed on α‐sarcomeric actin stained tissue sections, in fields where cells were perfectly longitudinal, showed nuclei centered in the cell body and intercalated discs were apparent. A line crossing the centre of nuclei was drawn and the length of the segment crossing the opposite intercalated discs was then measured, in analogy to what has been described in Vliegen *et al*.,^14^ and published by us recently.^15^

Vascular density was assessed by counting capillaries in at least 10 fields (200x magnification). Final data were expressed as the number of capillaries per mm^2^. Quantitative assessment of proliferation and apoptosis was carried out by counting the total number of Ki67, phosphorylated‐histone H3 and TUNEL‐positive cells in two complete cardiac sections per animal (630x magnification). Myocardial interstitial and perivascular fibrosis were identified using either Picrosirius Red or Azan Mallory (Sigma‐Aldrich) staining. Neutral lipid droplets were identified using Oil Red O (ORO) 2% (Sigma‐Aldrich) staining. At least 10 random fields were analysed (200x or 400x magnification). Areas of positive staining were measured in pixels and expressed as a percentage of total tissue area. Perivascular fibrosis was expressed as previously described.^16^ For ORO, measurements were further validated in tissue extracts measuring the dye concentration at 490 nm.

For quantification of BPIFB4, SDF‐1 and myosin heavy chain isoform α (MyHC‐α) expression, the whole left ventricular area was imaged and analysed. For BPIFB4, multiple photographs were taken with an INCell Analyser 2200 microscope (GE Healthcare Inc.) and collated to re‐construct the whole left ventricle.

### Molecular analyses

#### RNA isolation and RT‐qPCR

Total RNA was isolated from flash‐frozen murine left ventricular myocardium using TRIzol (Thermo Fisher Scientific, UK). The TaqMan primer‐probes for mouse *Bpifb4* and *Col1a1* were obtained from Thermo Fisher Scientific. Analysis was performed using the 2^‐ΔΔCt^ method with results normalised to β‐actin (*Actb*) internal control gene.

#### Protein extraction and western blotting

Proteins were extracted in RIPA buffer supplemented with a cocktail of proteases and phosphatases inhibitors (all from Sigma) and concentration was determined using the BCA Protein Assay Kit (Thermo Fisher Scientific). Primary antibodies (online supplementary *Table S3*) were incubated for 16 h at 4°C. Either β‐actin or β‐tubulin were used as loading control. Anti‐rabbit or ‐mouse IgG HRP‐conjugated were employed as secondary antibodies (both 1:5000, GE Healthcare). Membrane development was performed by an enhanced chemiluminescence‐based detection method (ECL™ Prime Western Blotting Detection Reagent, GE Healthcare) in a ChemiDoc‐MP system (Bio‐Rad). No more than one stripping procedure was performed on an individual membrane (RestoreTM Plus Western Blot Stripping Buffer, Thermo Fisher Scientific).

### Studies on human cells

The study was approved by the Ethics Committee of IRCCS Neuromed (No. 20160106–1006) and conducted in accordance with the Declaration of Helsinki. All participants gave written informed consent. Peripheral blood mononuclear cells from diabetic patients were isolated over Ficoll–Hypaque gradients (lymphocyte separation medium; MP Biomedicals, Aurora, OH, USA).

#### Flow cytometry detection of stromal cell‐derived factor‐1 positive mononuclear cells following exposure to WT‐BPIFB4 or LAV‐BPIFB4 protein

For intracellular detection of SDF‐1 positive cells, peripheral blood mononuclear cells were maintained in culture for 48 h in the presence or absence of either WT‐ or LAV‐BPIFB4 protein (18 ng/mL) produced as previously described.^17^ For detection of SDF‐1, a preliminary immunostaining of surface lineages markers (CD3, CD14, CD16, CD19, CD56, CD66) was performed. After 20 min incubation at 4°C in the dark, cells were washed and resuspended in PBS for the intracellular staining using the BD Cytofix/Cytoperm™ Fixation and Permeabilization Solution (BD Biosciences) according to the manufacturer's instructions. Cells were washed and resuspended in staining buffer (PBS + 2% foetal bovine serum). For each experimental condition, approximately 1*10^6^ cells were analysed using a FACS Verse Flow Cytometer (BD Biosciences). Antibodies are reported in online supplementary *Table S4*).

### Statistical analysis

Data are shown as individual values and mean ± standard error of the mean, with categorical variables as percentages. Comparisons were made using the Student's *t*‐test and one‐ or two‐way analysis of variance (ANOVA), as appropriate. The Brown–Forsythe test was used to determine equal variance between groups. Post‐hoc analysis of ANOVA included Tukey and Welch tests, as appropriate.

## Results

### BPIFB4 expression in the failing human heart

**Figure 1:**
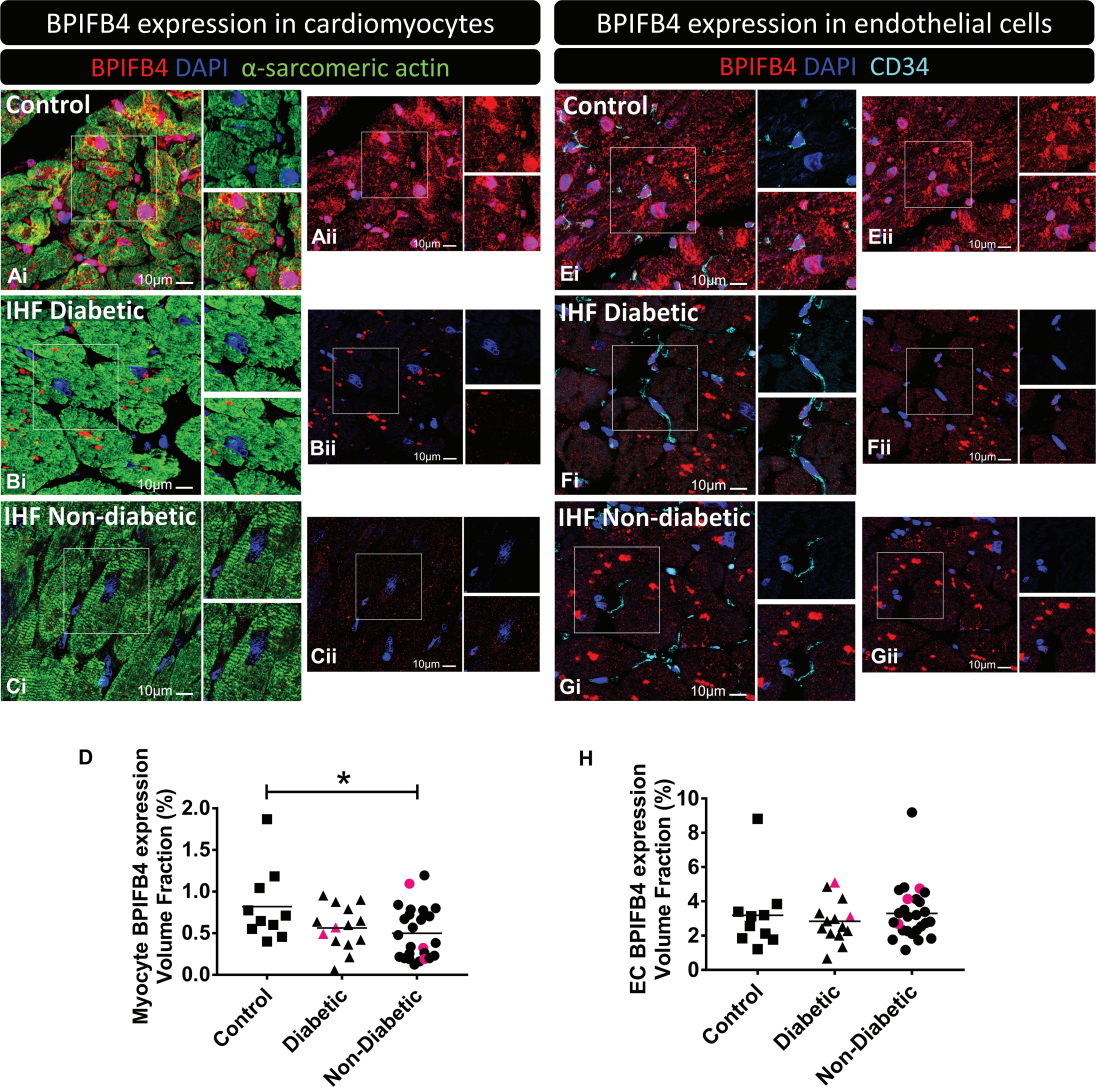
BPIFB4 expression in human cardiac samples. Samples were obtained from control hearts (*A,E*, control) and patients with ischaemic heart failure (IHF) with diabetes (*B,F*) or without (*C,G*). *(A–D)* BPIFB4 expression in cardiomyocytes. Fluorescence microscopy images show BPIFB4 in red fluorescence, cardiomyocytes α‐sarcomeric actin in green fluorescence, nuclei in blue (DAPI). (*E–H*) BPIFB4 expression in endothelial cells. BPIFB4 is in red fluorescence, CD34^+^ endothelial cells are shown with cyan pseudocolour, nuclei blue. All scale bars = 10 μm. The BPIFB4 channel (red) is provided also separately with the microphotographs labelled as (ii) for a better observation of the positive staining. In (*D,H*) data indicate the expression of BPIFB4 in cardiomyocytes (*D*) and endothelial cells (*H*) calculated as the positive volume fraction (percent of BPIFB4 positive staining in either cardiomyocytes or endothelial cells). In (*D*): *n* = 10 control, 14 diabetic, and 26 non‐diabetic. In (*H*): *n* = 10 control, 14 diabetic, and 26 non‐diabetic. Data are shown as individual values and mean. *P < 0.05 vs. controls. Red symbols correspond to carriers of the *LAV‐BPIFB4* variant.

In human hearts, immunoreactive BPIFB4 was detected in both cardiomyocytes and endothelial cells (*Figure* 1). BPIFB4 expression was reduced in cardiomyocytes of failing hearts, with 13 out 40 (32.5%) samples showing values below the lower limit of the control group. There was no difference between groups regarding the expression of BPIFB4 in coronary endothelial cells.

### Forced expression of *LAV‐BPIFB4* in non‐diabetic mice

In study 1, we sought to determine if the expression of the *LAV‐BPIFB4* transgene can alter normal heart function in young adult mice (protocol and results shown in online supplementary *Figure S1* *A* and supplementary *Table S5*). No change in echocardiography indices was observed in the *LAV‐BPIFB4*‐treated group, except an increase in the diastolic filling index E/A (*P* = 0.02 vs. vehicle).

### Validation of the diabetic murine model

In line with previous studies reviewed,^18^ db/db mice showed overt glycosuria [>2000 (110) mg/dL (mmol/L), data not shown] and excess body weight at 8 to 13 weeks of age (online supplementary *Figure S2* *A*). High levels of C‐peptide (*P* = 0.02 vs. controls) and insulin (*P* = 0.0006 vs. controls) were detected in peripheral blood at termination. Reduced 18F‐FDG uptake by the heart (56%, *P* = 0.02 vs. controls) confirmed a state of myocardial insulin resistance typical of the model (online supplementary *Figure S2* *B–D*).^19^ Moreover, the CT scan showed a remarkable increase of the subcutaneous fat pad (online supplementary *Figure S2* *D*).

Echocardiography validated the detrimental effect of diabetes on cardiac function (online supplementary *Table S6* and *Figure* *S2* *E–G*). At the 13‐week assessment, diastolic abnormalities were illustrated by a reduction in the E/A ratio (14%, *P* = 0.009 vs. controls) and mitral valve deceleration (25%, *P* = 0.036 vs. controls), and an increase in deceleration time (22%, *P* = 0.006 vs. controls). Fractional shortening (FS) and left ventricular ejection fraction (LVEF) declined from 8 to 13 weeks (3.8 and 4.1 percentage points, respectively, *P* < 0.05 for both comparisons), with both contractility parameters being depressed in 13‐week‐old diabetic mice compared with age‐matched controls (FS, 5.9 percentage points *P* = 0.004; LVEF, 6.5% points, *P* = 0.006). Hair re‐growth in the area depilated to perform echocardiography was reduced in the diabetic group (*P* < 0.001 vs. controls; online supplementary *Figure S2* *H*).

As shown in online supplementary *Figure S3* *A*, cardiomyocytes of diabetic mice showed a 1.13‐fold increase in cross‐sectional area (*P* = 0.04 vs. controls), but no change in length (*P* = 0.18; data not shown). Down‐regulation of MyHC‐α may contribute to impaired cardiac performance in diabetes.^20^, ^21^ MyHC‐α immunostaining was reduced by 50% in the heart of diabetic mice (*P* = 0.04 vs. controls; online supplementary *Figure S3* *B*). Moreover, the ratio between the MyHC‐α and MyHC‐β isoforms was reduced in the heart of diabetic mice, as assessed by western blot analysis (*P* < 0.01 vs. controls; online supplementary *Figure S3* *C*). As shown in online supplementary *Figure S3* *D–H*, diabetes reduced capillary density (*P* < 0.0001), while increasing interstitial (*P* = 0.04) and perivascular myocardial fibrosis (*P* < 0.001), collagen, type I, α1 expression (*P* = 0.03), and lipid content (*P* = 0.002). The staining for BPIFB4 did not differ in the heart of diabetic and control mice (online supplementary *Figure S3* *I*). Similarly, no difference between groups was observed regarding the ANP protein levels in the heart and immunoreactive ANP concentration in blood (online supplementary *Figure S3* *J–K*). This is in line with previous publications which show that cardiac ANP is unaltered, or even reduced, in obesity models like db/db mice,^22^, ^23^, ^24^ and that circulating ANP is rapidly eliminated by clearance receptors in adipocytes.^25^

### *BPIFB4* gene therapy in diabetic mice

**Figure 2:**
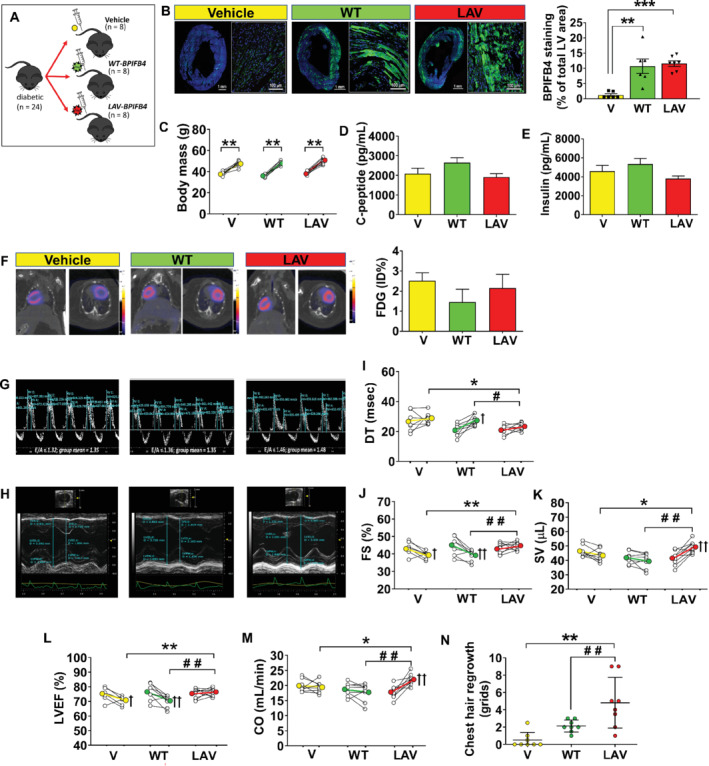
*LAV‐BPIFB4* gene therapy improves cardiac function in type 2 diabetic mice. (*A*) Diagram of mice randomisation (*n* = 8 mice per group). (*B*) Immunohistochemistry of BPIFB4 (green fluorescence) in the whole left ventricle (bar 1 mm) and a magnified section (bar 100 μm). Bar graph showing individual values and mean ± SEM of positive staining in the three groups. **P < 0.01, ***P < 0.001 vs. the vehicle group (V). (*C*) Body weight assessed at 8 and 13 weeks. Individual and mean values. **P < 0.01 vs. 8 weeks. (*D–E*) Peripheral blood levels of C‐peptide and insulin (mean ± SEM). (*F*) Representative images and bar graph (mean ± SEM) of 18F‐fluorodeoxyglucose (FDG) positron emission tomography/computed tomography imaging [values expressed as percent of injected dose (ID) per gram of tissue; *n* = 3 per group]. (*G–M*) Graphs showing results of echocardiography assessments at 8 and 13 weeks. Representative images of E and A waves measured using pulsed‐wave Doppler (*G*) and M‐mode traces (*H*). Graphs showing the improvement induced by *AAV‐LAV‐BPIFB4* (LAV) on indices of diastolic and systolic function: (*I*) deceleration time (DT), (*J*) fractional shortening (FS), (*K*) stroke volume (SV), (*L*) left ventricular ejection fraction (LVEF), (*M*) cardiac output (CO). Data expressed as individual values and mean ± SEM; *n* = 8 mice per group. *P < 0.05 and **P < 0.01 vs. vehicle (V), ^#^ *P* < 0.05 and ^##^ *P* < 0.01 vs*. AAV‐WT‐BPIFB4* (WT), ^+^ *P* < 0.05 and ^++^ *P* < 0.01 vs. 8 weeks within the same group. (*N*) The degree of hair re‐growth determined at 13 weeks in the chest area depilated for performing echocardiography. A 9‐block grid system was used, with hair growth within grids scored 1–9, with a further score ^9^ for mice with complete hair re‐growth. Graph showing individual values and mean ± SEM in the three groups. **P < 0.01 vs. vehicle (V) and ^##^ *P* < 0.01 vs*. AAV‐WT‐BPIFB4* (WT).

We next investigated if forced expression of the human *BPIFB4* gene protects the heart from diabetes‐induced damage, and if *LAV‐BPIFB4* is superior to *WT‐BPIFB4* in this respect (protocol in *Figure* 2 *A* and online supplementary *Figure S1* *B*).

The liver‐specific thyroxine‐binding globulin (TBG) promoter was adopted for the AAV assembling.^6^ As a confirmation, GFP fluorescence was detected in the liver (online supplementary *Figure S4*. *A–F*), but not in the heart (online supplementary *Figure S4*. *G–J*), of mice injected with the AAV‐GFP vector. Using an antibody that recognises both the murine and human protein, we demonstrated that *WT‐BPIFB4* and *LAV‐BPIFB4* gene transfer induced similar increases in BPIFB4 staining in the heart, with specific localisation of the fluorescent signal in cardiomyocytes (*P* < 0.01 vs. vehicle; *Figure* 2 *B*). Using RT‐qPCR and human‐specific primers, validated on human coronary artery endothelial cells (data not shown), we could not detect any *BPIFB4* mRNA transcript in the heart of mice given gene therapy (either *WT‐BPIFB4* or *LAV‐BPIFB4*). Additionally, RT‐qPCR with murine‐specific primers showed similar *Bpifb4* transcript levels in the heart of vehicle and *WT‐BPIFB4*‐ or *LAV‐BPIFB4‐*treated groups (online supplementary *Figure S5* *A*). Together, these data suggest that (i) the liver, but not the heart, was transduced by the viral vector, and (ii) the up‐regulation of BPIFB4 in cardiomyocytes of *WT‐BPIFB4*‐ or *LAV‐BPIFB4‐*treated mice was likely due to an uptake of circulating protein and not to an induction of the endogenous murine gene. These data fit with the increased BPIFB4 protein levels detected in monocytes of *LAV‐BPIFB4*‐treated ApoE knockout mice, without any mRNA upregulation (either human or murine).^8^ To verify that the transgenic protein was also present in the plasma fraction, we measured BPIFB4 using a human‐dedicated ELISA on samples collected 4 weeks after gene therapy. The assay detected the protein in plasma of the vehicle group, thus suggesting cross‐reactivity of the antibody for murine Bpifb4. Consequently, the finding that *BPIFB4*‐transduced mice did not show increased plasma levels compared with vehicle should be considered with caution (online supplementary *Figure S5* *B*).

Neither *WT‐BPIFB4* nor *LAV‐BPIFB4* affected body weight or metabolic control, with both groups showing glycosuria (>2000 mg/mL), and high C‐peptide and insulin levels in peripheral blood (*Figure* 2 *C–E*). Moreover, the 18F‐FDG uptake by the heart was similar in all groups (*Figure* 2 *F*).

Randomised groups were well balanced for basal echocardiography parameters, whereas there were significant differences attributable to the treatments (*Figure* 2 *H–M* and online supplementary *Table S6*). *LAV‐BPIFB4* improved diastolic function, preventing the increase in mitral valve deceleration time seen in the other groups (*Figure* 2 *G–I*). Dimensional assessment denoted no effect of treatments on left ventricular mass, whereas *LAV‐BPIFB4*, but not *WT‐BPIFB4*, prevented the increase in end‐systolic volume seen from 13 to 18 weeks in diabetic mice. Moreover, *LAV‐BPIFB4*‐treated mice showed improved systolic function compared with vehicle or *WT‐BPIFB4* groups (*Figure* 2 *H–M*). Both stroke volume and cardiac output increased in the *LAV‐BPIFB4* group from baseline to final measurements (17% and 23%, respectively, *P* < 0.01 for both comparisons). These changes could not be ascribed to differences in heart rate, which was similar among groups at baseline and final measurements (online supplementary *Table S6*). An accelerated hair re‐growth was observed in mice treated with *LAV‐BPIFB4* (*P* < 0.05 vs. vehicle), but not in those given *WT‐BPIFB4* (*Figure* 2 *N*).

**Figure 3:**
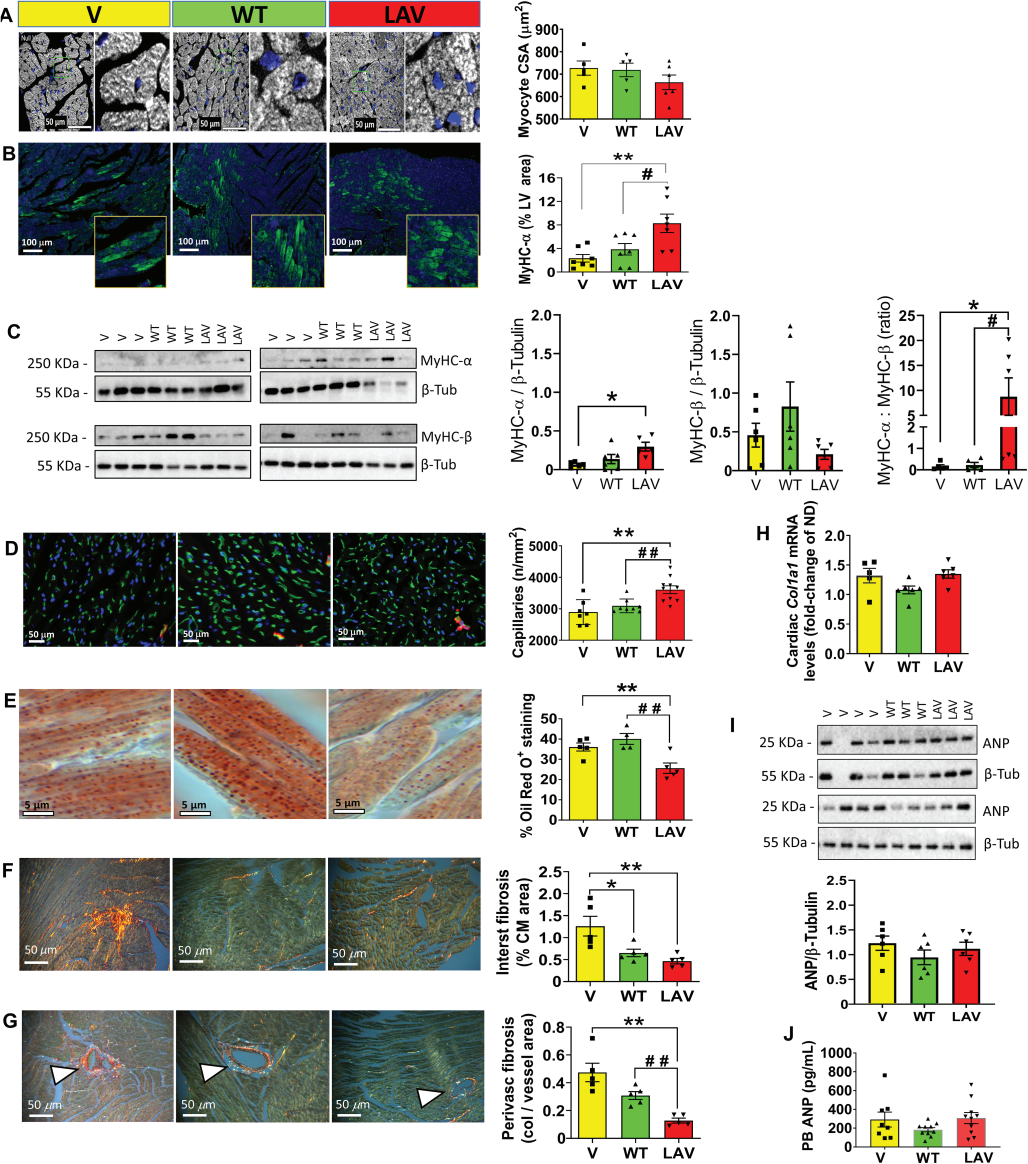
*LAV‐BPIFB4* promotes cardiomyocyte expression of contractile protein and prevents capillary rarefaction, lipid accumulation and fibrosis. (*A*) Representative fluorescence images showing areas of transversally sectioned myocardium. Cardiomyocytes identified by α‐sarcomeric actin staining (white pseudocolour) and nuclei labelled by DAPI. Scale bars: 50 μm. Right panels are higher magnifications of the areas delimited by a green outline. Graph shows morphometric data of cardiomyocyte cross‐sectional area (CSA) from individual mice (*n* = 5 to 6 per group) together with mean ± SEM. (*B*) Representative fluorescence images showing the expression of myosin heavy chain isoform alpha (MyHC‐α). Scale bar: 100 μm. Graph with individual values (*n* = 6 per group) together with mean ± SEM. **P < 0.01 vs. vehicle, ^#^ *P* < 0.05 vs. WT. (*C*) Western blot analysis confirming the induction of MyHC‐α and increased ratio between MyHC‐α and MyHC‐β. Individual values (*n* = 6 per group) with mean ± SEM. *P < 0.05 vs. vehicle, ^#^ *P* < 0.05 vs. WT. (*D*) Myocardial microvasculature. Representative images of isolectin B4 (green) positive endothelial cells and α‐smooth muscle actin (red) positive smooth muscle cells, with nuclei identified by DAPI (blue). Scale bars: 50 μm. Graph showing the relative abundance of capillaries (*n* = 7 to 10 mice per group). Individual values and mean ± SEM. **P < 0.01 vs. vehicle, ^##^ *P* < 0.01 vs. WT. (*E*) Lipid accumulation. Representative images of Oil red O staining and graph showing morphometric data of lipid accumulation expressed as percentage of the area section. Scale bars: 5 μm. Individual values and mean ± SEM. *n* = 4 to 5 mice per group. **P < 0.01 vs. vehicle, ^##^ *P* < 0.01 vs. WT. (*F,G*) Interstitial (*F*) and perivascular (*G*) fibrosis (triangles point to blood vessels). Representative images of picrosirius red staining and graph showing morphometric data of fibrosis. Scale bars: 50 μm. Individual values and mean ± SEM; *n* = 5 mice per group. *P < 0.05 and **P < 0.01 vs. vehicle, ^##^ *P* < 0.01 vs. WT. (*H*) Cardiac Col1a1 mRNA individual values and mean ± SEM; *n* = 6 per group. (*I*) Western blot of cardiac atrial natriuretic peptide (ANP). Individual values and mean ± SEM; *n* = 6 per group. (*J*) Immunoreactive ANP levels in peripheral blood. Individual values and mean ± SEM; *n* = 8 to 10 per group.

Neither *WT‐BPIFB4* nor *LAV‐BPIFB4* influenced cardiomyocyte size (*Figure* 3 *A*) or apoptosis, as assessed by TUNEL staining (data not shown). Although Ki67^+^ cardiomyocytes were more frequent in the *LAV‐BPIFB4* group (*P* < 0.05 vs. vehicle, online supplementary *Figure S6* *A*,*B*), phosphorylated histone H3 expression was very rare (online supplementary *Figure S6* *C*), thus ruling out an effect of gene therapy on proliferation. Importantly, *LAV‐BPIFB4*, but not *WT‐BPIFB4*, increased the expression of MyHC‐α in cardiomyocytes, together with the ratio between the α and β MyHC isoforms in the heart (*P* < 0.05 vs. vehicle for both comparisons, *Figure* 3 *B*,*C*). In addition, *LAV‐BPIFB4* gene therapy increased the density of capillaries, while reducing lipid content, and interstitial and perivascular fibrosis (*P* < 0.01 vs. vehicle for all comparisons; *Figure* 3 *D‐G*). The reduction in fibrosis was likely due to enhanced collagen degradation, since cardiac *Col1a1* mRNA levels were not affected by *LAV‐BPIFB4* gene therapy (*Figure* 3 *H*). Likewise, *LAV‐BPIFB4* did not affect the levels of ANP in the heart and peripheral blood (*Figure* 3 *I*,*J*).

### Implication of the SDF‐1/CXCR4 in LAV‐BPIFB4‐induced cardiac protection

**Figure 4:**
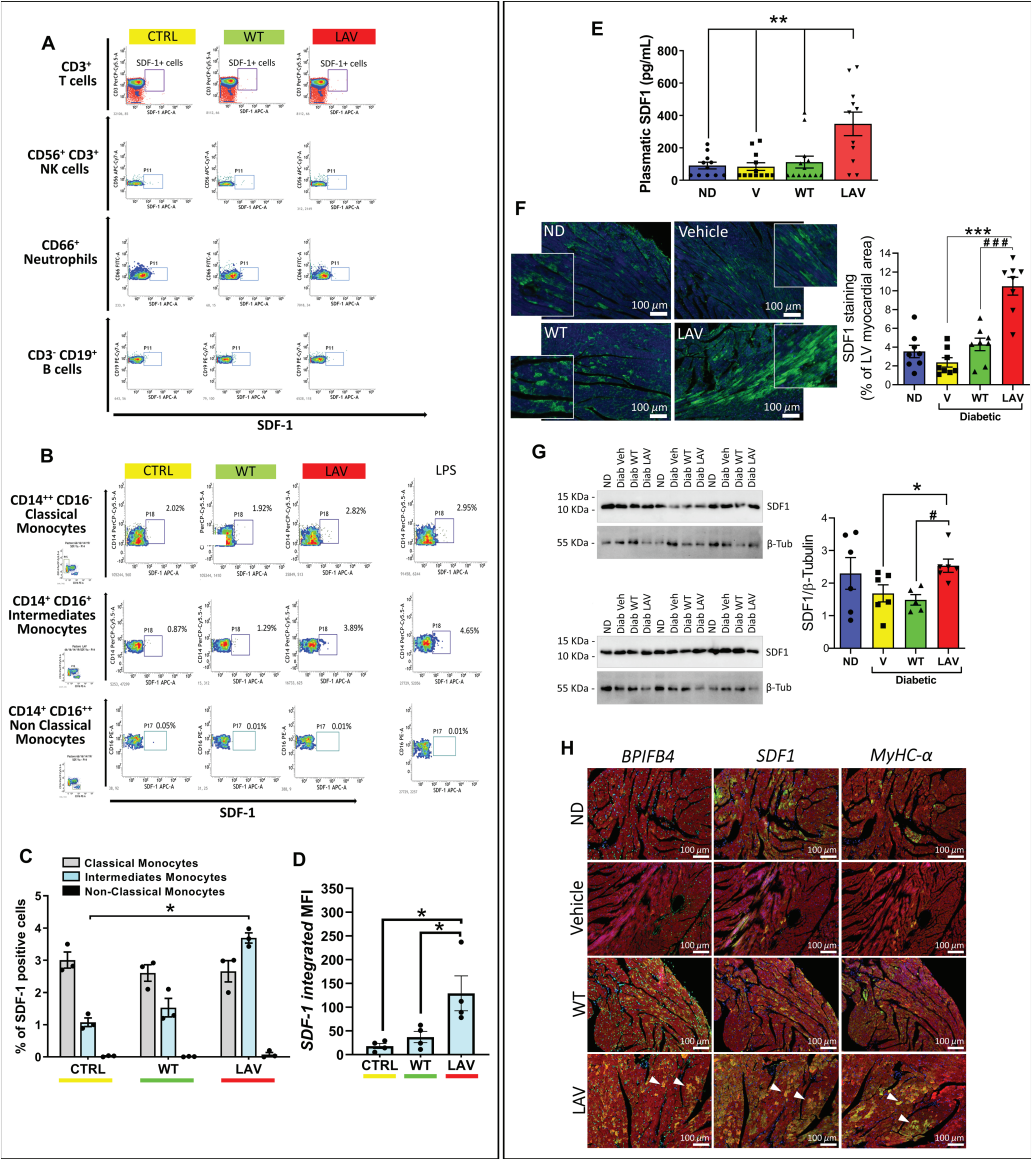
LAV‐BPIFB4 induces stromal cell‐derived factor‐1 (SDF‐1) in human monocytes *in vitro* and in peripheral blood and hearts of diabetic mice *in vivo*. (*A–D*) Cytofluorimetric analysis of the peripheral blood mononuclear cells (PBMCs) from type 2 diabetic patients incubated with WT‐BPIFB4 or LAV‐BPIFB4 protein or vehicle for 48 h. LPS was used as a positive control stimulation. At the end of the cell culture treatment, PBMCs were recovered, stained for the different antigens and anti‐intracellular SDF‐1, and analysed by flow cytometry. (*A*) T cells, NK cells, neutrophils, and B cells. (*B*) Monocytes. (*C*) Frequency of SDF‐1 positive events within subclasses of monocytes. (*D*) Relative fluorescence intensity of SDF‐1 in the intermediate monocyte population. Individual values and mean ± SEM. *n* = 4 biological replicates. *P < 0.05. (*E*) Immunoreactive SDF‐1 levels in murine peripheral blood. Bar graphs show individual values and mean ± SEM. *n* = 11–13 mice per group. **P < 0.001 between longevity‐associated variant (LAV) and other groups. (*F*) Immunohistochemistry (SDF‐1 in green, DAPI in blue, scale bars: 100 μm) and (*G*) western blot analyses of SDF‐1 expression in the mice heart. Bar graphs show individual values and mean ± SEM. *n* = 5–8 mice per group. *P < 0.05, ***P < 0.001 vs. vehicle; ^#^ *P* < 0.05, ^###^ *P* < 0.001 vs. WT. (*H*) Immunohistochemistry (scale bars: 100 μm) of BPIFB4, SDF‐1 and myosin heavy chain isoform alpha (MyHC‐α) expression (all in green), α‐sarcomeric actin in red, and DAPI blue. Triangles indicate cardiomyocytes co‐expressing the three proteins.

The SDF‐1/CXCR4 duo is reportedly involved in *LAV‐BPIFB4* capacity to induce M2 macrophage polarisation in an atherosclerotic model.^8^ New results from flow cytometry analysis of circulating mononuclear cells from type 2 diabetic patients indicate that *in vitro* stimulation with the LAV‐BPIFB4 protein selectively induced the expression of SDF‐1 in CD14^+^CD16^+^ intermediate monocytes, but not in other leukocyte populations (*Figure* 4 *A–D*).

Moreover, we gathered new evidence supporting a role of SDF‐1 in *LAV‐BPIFB4*‐induced protection against diabetic cardiomyopathy. *LAV‐BPIFB4*‐treated mice had higher levels of circulating SDF‐1 compared with the *WT‐BPIFB4* and vehicle group (*P* < 0.01 for both comparisons; *Figure* 4 *E*). Western blot analysis and immunostaining of left ventricular tissue showed that *LAV‐BPIFB4*, but not *WT‐BPIFB4*, increased SDF‐1 expression in the heart of diabetic mice (*Figure* 4 *F–G*). Staining of consecutive sections identified the concurrent upregulation of BPIFB4, SDF‐1 and MyHC‐α in cardiomyocytes of *LAV‐BPIFB4*‐treated mice (*Figure* 4 *H* and online supplementary *Figure S7*).

**Figure 5:**
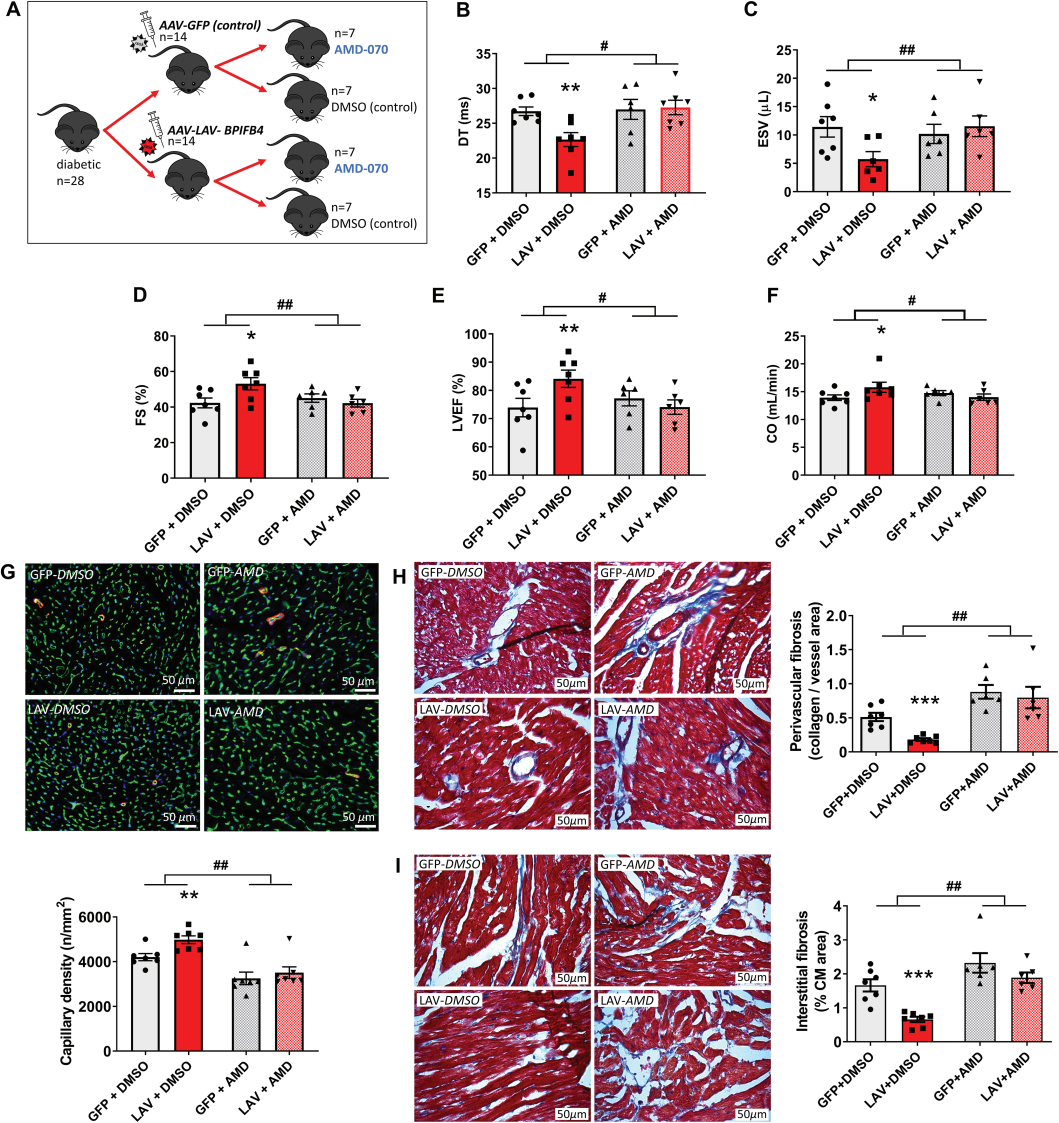
Inhibition of the effects of *LAV‐BPIFB4* gene therapy by an antagonist of the CXCR4 receptor. (*A*) Experimental protocol: seven mice entered the study but only six in each antagonist group were considered because compliant in taking the medicated jelly. All data shown as individual values and means ± SEM. (*B–F*) Results of echocardiography. The antagonist inhibits the effect of *LAV‐BPIFB4* gene therapy on deceleration time (DT) (*B*), end‐systolic volume (ESV) (*C*), fractional shortening (FS) (*D*), left ventricular ejection fraction (LVEF) (*E*), and cardiac output (CO) (*F*). *P < 0.05 and **P < 0.01 vs. GFP + DMSO, ^#^ *P* < 0.05 and ^##^ *P* < 0.01 in comparison with changes between LAV + DMSO and GFP + DMSO. (*G*) Capillary density (scale bars: 50 μm). Isolectin B4 (green) identifies endothelial cells, α‐smooth muscle actin (red), and DAPI nuclei (blue). Bar graph showing the results. **P < 0.01 vs. GFP + DMSO, ^##^ *P* < 0.01 in comparison with changes between LAV + DMSO and GFP + DMSO. (*H,I*) Cardiac interstitial (*H*) and perivascular (*I*) fibrosis (blue) analysed by Azan Mallory staining (scale bars: 50 μm). ***P < 0.001 vs. GFP + DMSO, ^##^ *P* < 0.01 in comparison with changes between LAV + DMSO and GFP + DMSO.

To establish a cause–effect relationship, diabetic mice were randomised to receive *LAV‐BPIFB4* or *GFP* in combination with the orally active CXCR4 antagonist, AMD‐070, or its vehicle, DMSO (*Figure* 5 *A* and online supplementary *Figure S1* *B*). Of the 16 mice entering the AMD‐070 treatment, two (one in the *LAV‐BPIFB4* and the other in the *GFP* group) were excluded because of not taking the medicated jelly. The effective inhibition of downstream signalling by AMD‐070 was confirmed by assessing the phosphorylation/activation of Erk1/2 in the heart (online supplementary *Figure S8* *A*,*B*). This study provided two important results. First, it confirmed *LAV‐BPIFB4* gene therapy improved functional and structural endpoints in comparison with a control group injected with the AAV‐*GFP*, thus excluding a background effect of the viral vector used to transfer the *LAV‐BPIFB4* sequence. Second, the CXCR4 antagonist precluded *LAV‐BPIFB4* benefits on echocardiographic, histological, and expressional endpoints (*Figure* 5 and online supplementary *Table S7*). This included the repression of *LAV‐BPIFB4*‐induced increase in MyHC‐α and MyHC‐α to MyHC‐β ratio (online supplementary *Figure S8* *C*,*D*).

### *LAV‐BPIFB4* improves vascular function in diabetic mice

**Figure 6:**
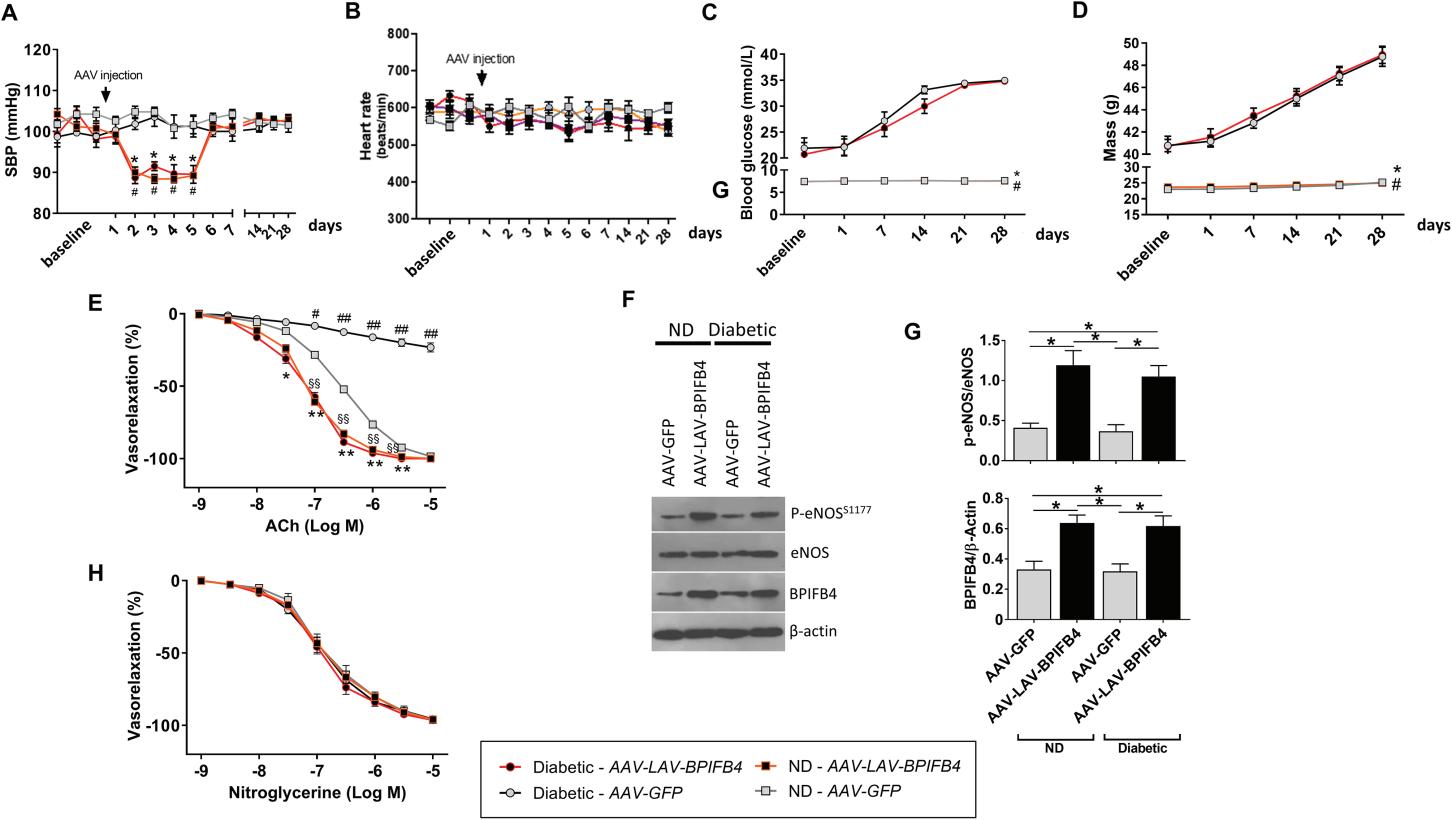
*LAV‐BPIFB4* gene therapy benefits vascular function in diabetic mice (study 3). (*A*) Systolic blood pressure (SBP) in non‐diabetic (ND) mice and obese diabetic mice treated with *AAV9‐LAV‐BPIFB4* or *AAV9‐GFP* (*n* = 5 to 6 per group). Arrow indicates gene therapy delivery time point. *P < 0.05 diabetic vs. diabetic given *AAV9‐GFP*; ^#^ *P* < 0.05 ND vs. ND given *AAV9‐GFP*. (*B*) Heart rate data in the same animals. (*C,D*) Graphs show blood glucose levels (*C*) and body mass (*D*) before and after treatment with *AAV9‐LAV‐BPIFB4* or *AAV9‐GFP*. *P < 0.05 vs. diabetic given *AAV9‐GFP* and ^#^ *P* < 0.05 vs. diabetic given *AAV9‐LAV‐BPIFB4*. (*E–H*) Assays performed on mesenteric arteries excised 4 weeks after gene therapy. (*E*) Graphs show the dose–response curves to acetylcholine (ACh, 10^−9^ M to 10^−5^ M), *n* = 5 experiments. *P < 0.05 and **P < 0.01 vs. ND given *AAV9‐GFP*; ^§^ *P* < 0.05 and ^§§^ *P* < 0.01 vs. ND given *AAV9‐GFP*; ^#^ *P* < 0.05 and ^##^ *P* < 0.01 vs. all. (*F*) Representative western blot of mesenteric arteries obtained from ND and diabetic mice treated with *AAV9‐LAV‐BPIFB4* or *AAV9‐GFP*. (*G*) Bar graphs show optical density quantification. *n* = 3 experiments. Individual values and means ± SEM. *P < 0.05. (*H*) Dose–response curves to nitroglycerine (10^−9^ M to 10^−5^ M). *n* = 5 experiments. All data are given as means ± SEM.

The protocol assessing systemic haemodynamics and endothelial function is illustrated in online supplementary *Figure S1* *C*. *LAV‐BPIFB4* caused a temporary reduction in systolic blood pressure in diabetic and non‐diabetic mice, with pressure values returning to baseline by day 6 until the end of the study (*Figure* 6 *A*). No treatment effect was observed on heart rate throughout the study duration (*Figure* 6 *B*). Plasma glucose levels and body mass were not different in mice given *LAV‐BPIFB4* or control viral vector (*Figure* 6 *C*,*D*). Moreover, *LAV‐BPIFB4* improved the diabetes‐induced impairment in endothelial‐dependent vasorelaxation and eNOS phosphorylation (*Figure* 6 *E–G*), without affecting vasorelaxation by nitroglycerine (*Figure* 6 *H*). Taken together, these results indicate that although the haemodynamic effect of *LAV‐BPIFB4* vanished after 1 week, the benefit on endothelial‐mediated vasorelaxation persisted up to a month after treatment, which is in line with our previous report in aged mice.^5^

Finally, we assessed if vessels transfected with *LAV‐BPIFB4* are protected from high glucose‐induced endothelial dysfunction through a CXCR4‐mediated mechanism. In line with this, *ex vivo LAV‐BPIFB4*‐transfected vessels were responsive to acetylcholine‐evoked vasorelaxation (online supplementary *Figure S9*), which was not observed using empty‐ or *WT‐BPIFB4*‐containing vectors (online supplementary *Figure S9*). Moreover, treatment with AMD3100, a selective CXCR4 receptor inhibitor, abolished the benefit of *LAV‐BPIFB4* on vascular function (online supplementary *Figure S9*), thus supporting the involvement of CXCR4 signalling.

## Discussion

In the present study, ischaemic heart failure was associated with an increased incidence of BPIFB4 down‐regulation in cardiomyocytes as assessed by immunohistochemistry, with no additional effect being seen in the diabetic group. *LAV‐BPIFB4* homozygosity was detected at the expected frequency, but carriers were too few to allow associative assessments.

Next, we sought to determine if *LAV‐BPIFB4* therapy can prevent the early progression of cardiomyopathy in a model of type 2 diabetes. The study was completed with no loss to follow‐up and no evidence of adverse events. Interestingly, *LAV‐BPIFB4*, but not *WT*‐*BPIFB4*, exerted therapeutic effects, despite both appearing to fail in achieving metabolic control. General inspection denoted an accelerated hair re‐growth in *LAV‐BPIFB4*‐treated animals, which may be compatible with improvements in dermal microangiopathy and follicle dysfunction.

Both WT‐BPIFB4 and LAV‐BPIFB4 were expressed by the heart, likely via uptake from the circulation. However, only the latter had salutary effects on left ventricular function in diabetic mice. Moreover, *LAV‐BPIFB4* prevented capillary rarefaction, fibrosis and lipid accumulation in the diabetic heart. Reduced fibrosis may account for the improved diastolic function. Regarding factors influencing systolic function, we excluded an effect of *LAV‐BPIFB4* on cardiomyocyte hypertrophy, proliferation, or apoptosis. Moreover, *LAV‐BPIFB4* therapy transitorily reduced blood pressure (without affecting heart rate), with this temporary effect not leading to left ventricular mass changes. Interestingly, *LAV‐BPIFB4* rescued the abundance of cardiomyocytes expressing MyHC‐α, improved endothelial function, and enhanced vascular eNOS phosphorylation in diabetic mice, as seen previously in hypertensive rats.^5^ The work performed by the heart during ejection is dependent upon the inherent contractile property of individual myocytes, which in turn is strictly related to the reciprocal expression of MyHC isoforms.^26^ MyHC‐α is reportedly down‐regulated in the diabetic heart^20^; while small amounts of MyHC‐α expression are sufficient to aid power output in cardiomyocytes.^21^ Therefore, induction of the contractile protein and restoration of endothelial function may represent pivotal drivers in the improved systolic performance we have seen in *LAV‐BPIFB4*‐treated mice.

Controversy surrounds the benefit of cardiovascular treatments targeting SDF‐1 signalling. SDF‐1 supplementation in the form of protein, plasmid or engineered cell therapy, reportedly improved cardiac function in mice with ischaemic cardiomyopathy.^27^, ^28^ However, the STOP‐HF trial, using a single endocardial administration of plasmid SDF‐1 in patients with ischaemic heart failure, failed to demonstrate its primary endpoint of improved composite score.^27^ Blockade of SDF‐1 signalling using a CXCR4 antagonist reduced cardiac fibrosis in diabetic rats.^29^ Moreover, SDF‐1 has pro‐angiogenic activity,^30^ but is thought to exert negative inotropic actions through the CXCR4 receptor.^31^

In our study, *LAV‐BPIFB4*, but not *WT‐BPIFB4* therapy, increased SDF‐1 levels in peripheral blood and heart of db/db mice. In addition, treatment of human diabetic mononuclear cells with the LAV‐BPIFB4 protein, but not WT‐BPIFB4, induced intermediate monocytes to express SDF‐1. Noteworthy, myeloid cells showed the capacity to uptake BPIFB4 in mice treated with *LAV*‐*BPIFB4* through a mechanism that remains unknown.^8^ Importantly, the use of two different CXCR4 antagonists confirmed that SDF‐1 signalling is involved in the therapeutic effects of *LAV‐BPIFB4*, including prevention of contractile dysfunction, possibly through induction of MyHC‐α and a reduction in myocardial fibrosis, microangiopathy, and endothelial dysfunction.

In conclusion, this is the first demonstration for horizontal transfer of a LAV gene to produce functional and anatomical benefits on the diabetic heart (online supplementary *Figure S10*).

### Perspectives and study limitations

The study of BPIFB4 expression in human hearts was limited to the use of immunohistochemistry. Unavailability of frozen left ventricular material precluded the possibility to confirm the data employing a different technique, such as western blot. Therefore, additional studies are necessary to determine if BPIFB4 down‐regulation in the human heart contributes to the development of cardiomyopathies, and thereby may represent a useful diagnostic and therapeutic biomarker.

Additional research is also warranted to determine the modality of the *BPIFB4* transgene delivery to the heart, the mechanism by which *LAV‐BPIFB4* induces SDF‐1 expression, the optimal *LAV‐BPIFB4* dosage, and the need to repeat *LAV‐BPIFB4* gene therapy for long‐term support of the diabetic heart.

Finally, it would be interesting to ascertain if additional cardiovascular improvements can be achieved through superimposing *LAV‐BPIFB4* gene therapy to available anti‐diabetic drugs. Non‐gene‐based approaches using a recombinant protein may also be considered for clinical translation.

### Funding

This work was supported by the British Heart Foundation (BHF) project grant ‘Longevity‐associated BPIFB4 gene therapy for treatment of ischemic disease’ (PG/15/54/31559); the BHF Centre for Regenerative Medicine Award (II) ‐ “Centre for Vascular Regeneration” (RM/17/3/33381);  the Italian Ministry of Health Ricerca Corrente for IRCCS Multimedica; the Young Investigator grant from the Italian Ministry of Health (GR‐2013‐02358692); the Cariplo Foundation grant ‘BPIFB4 isoforms: possible genetic risk factor and therapeutic tool for human frailty’; and the ‘HEARTzeimer’ grant from the Friuli Venezia Giulia Region. E. Ciaglia was supported by a fellowship from Fondazione Umberto Veronesi (FUV 2019 cod.2198).

**Conflict of interest**: A.A.P. and C.V. own shares of LGV1 Inc. and have filed a patent on the potential therapeutic use of LAV‐BPIFB4. The other authors have nothing to disclose.

## Supporting information


**Table S1.** Clinical characteristics of patients enrolled to the study.
**Table S2.** Immunofluorescence antibodies used on cardiac tissues.
**Table S3.** List of western blotting antibodies.
**Table S4.** List of flow‐cytometry antibodies.
**Table S5.** Study 1: echocardiography indices in non‐diabetic mice given a single systemic injection of *LAV‐BPIFB4* or vehicle.
**Table S6.** Echocardiography results of first part of study 2 assessing *LAV‐BPIFB4* vs. *WT‐BPIFB4* gene therapy in diabetic mice.
**Table S7.** Echocardiography results of second part of study 2 assessing the effect of SDF‐1 antagonism on *LAV‐BPIFB4* gene therapy.

**Figure S1.** Experimental in vivo protocols.

**Figure S2.** Comparison between diabetic and non‐diabetic mice at 8 and 13 weeks of age. (*A*) Body weight. (*B,C*) Bar graphs showing peripheral blood levels of C‐peptide (*B*) and insulin (*C*). (*D*) FDG uptake by the heart. (*E‐G*) Echocardiography parameters: E/A ratio (*E*), fractional shortening (*F*) and left ventricular ejection fraction (*G*). (*H*) Chest hair regrowth.

**Figure S3.** Comparison between diabetic and non‐diabetic mice at 13 weeks of age. (*A*) Myocyte cross‐sectional area. (*B*) Expression of MyHC‐α. (*C*) Western blot of the cardiac MyHC isoforms alpha and beta. (*D*) Capillary density. (*E,F*) Interstitial (*E*) and perivascular (*F*) fibrosis. (*G*) Cardiac *collagen 1A1* mRNA levels. (*H*) Oil red O showing lipid accumulation. (*I*) BPIFB4 expression. (*J*) Western blot of cardiac ANP. (*K*) Peripheral blood immunoreactive ANP levels.

**Figure S4.** (*A–F*) Representative immunohistochemistry images of livers transduced with *AAV9‐GFP*. An anti‐GFP antibody (*B*) was used to confirm the specificity of the green signal from GFP (*A*). Merged fluorescent signals (*C*). A control in which the primary antibody was omitted is shown in (*D–F*). (*G–J*) The heart of mice injected with *AAV9‐GFP* do not show any GFP expression (*G,H*). *AAV9‐LAV‐BPIFB4*‐transduced mice were used as controls (*I,J*).

**Figure S5.** Comparison between diabetic mice at 13 weeks of age. (*A*) Murine *Bpifb4* mRNA expression in the hearts. (*B*) Plasma levels of BPIFB4.

**Figure S6.** Proliferation of cardiomyocytes. (*A,B*) Abundance of Ki67‐positive cardiomyocytes. (*C*) Phosphorylated histone H3 staining (nuclear green fluorescence) showing very few positive cells expressing the marker.

**Figure S7.** Expression of BPIFB4, SDF‐1 and cardiac MyHC‐α in hearts of three diabetic mice treated with LAV‐BPIFB4.

**Figure S8.** Cell signalling activated by LAV‐BPIFB4 and antagonised by AMD‐070 in mice hearts. (*A*) Erk1/2 immunoblots. **(** *B* **)** Densitometry analysis. (*C*) Cardiac MyHC‐α and ‐β immunoblots. (*D*) Densitometry analysis.

**Figure S9.** Vessels transfected with LAV‐BPIFB4 are protected from high glucose‐induced endothelial dysfunction through a CXCR4‐mediated mechanism. (*A*) Graphs showing vasorelaxation induced by acetylcholine. (*B*) Western blot for endothelial nitric oxide synthase in the groups with or without AMD3100 treatment.

**Figure S10.** Translational perspective. Here, we present translational evidence for a novel salutary approach inspired by the successful case of centenarians, who escape major age‐related disease and vertically transmit genetic protection against disease to offspring. Our study demonstrates that horizontal transfer of a human longevity‐associated gene variant alleviates diabetic cardiomyopathy. The encoded protein could become a novel therapeutic product to extend health‐span in cardiovascular patients.
