# Supplementary material for: Transfer of a human gene variant associated with exceptional longevity improves cardiac function in obese type 2 diabetic mice through induction of the SDF‐1/CXCR4 signalling pathway
Source: Eur J Heart Fail. 2020 May 8;22(9):1568–81. doi: 10.1002/ejhf.1840 (PMC8220375; doi:10.1002/ejhf.1840)
Supplement: Supplementary file 1 — Table S1. Clinical characteristics of patients enrolled to the study. Table S2. Immunofluorescence antibodies used on cardiac tissues. Table S3. List of western blotting antibodies. Table S4. List of flow‐cytometry antibodies. Table S5. Study 1: echocardiography indices in non‐diabetic mice given a single systemic injection of LAV‐BPIFB4 or vehicle. Table S6. Echocardiography results of first part of study 2 assessing LAV‐BPIFB4 vs. WT‐BPIFB4 gene therapy in diabetic mice. Table S7. Echocardiography results of second part of study 2 assessing the effect of SDF‐1 antagonism on LAV‐BPIFB4 gene therapy. [file EJHF-22-1568-s005.docx]

**Transfer of a human gene variant associated with exceptional longevity improves cardiac function in obese type-2 diabetic mice through induction of the SDF-1/CXCR4 signalling pathway**

Zexu Dang *et al.*

**Supplementary Data**

**Supplementary Table I – Clinical characteristics of patients enrolled to the study**

|  | **Ischaemic**  **Heart FAILURE**  **All**  **(n=40)** | **Ischaemic**  **Heart FAILURE**  **Diabetic**  **(n=14)** | **Ischaemic**  **Heart FAILURE**  **Non-diabetic**  **(n=26)** | **Controls**  **(n=10)** | | ***p*** **value** | |
| --- | --- | --- | --- | --- | --- | --- | --- |
| **Age (years)** | 60±7 | 62±5 | 59±8 | | 43±13^a, b^ | | a <0.0001; b <0.0001 |
| **Sex (m/f)** | 35/5 | 12/2 | 23/3 | | 4/6 | | 0.005 |
| **LAV PBIFB4 homozygous** | 12.5% | 14.3% | 11.5% | | 10% | | N.S. |
| **Duration of disease (months)** | 136.5±105.9 | 142.7±92.9 | 133±114.5 | | - | | n.s. |
| **NYHA class iv (%)** | 35.0 | 44.4 | 31.2 | | - | | N.S. |
| **LV ejection fraction (%)** | 24±5 | 24±4 | 24±5 | | 54-74 (Normal Values) | | n.s. |
| **Gross anatomy** | | | | | | | |
| **Heart mass (grams)** | 514±130 | 566±141 | 486±117 | | 321±89 ^a, b^ | | a = 0.0009; b = 0.032 |
| **Transverse diameter (mm)** | 132±16 | 135±12 | 131±18 | | 99±21 ^a, b^ | | a = 0.0003; b = 0.0006 |
| **Inner longitudinal diameter (mm)** | 93±13 | 97±12 | 91±14 | | 71±8 ^a, b^ | | a = 0.0004; b = 0.004 |
| **Wall thickness (mm)** | | | | | | | |
| **LV ant** | 12±5 | 12±7 | 12±5 | | 10±1 | | n.s. |
| **LV lat** | 12±4 | 14±5 | 12±3 | | 10±1 | | n.s. |
| **LV post** | 9±4 | 9±5 | 9±4 | | 12±1 ^b^ | | b = 0.03 |
| **RV** | 6±2 | 6±2 | 6±2 | | 3±1 ^a, b^ | | a = 0.049; b = 0.0166 |
| **Septum** | 12±5 | 12±5 | 12±5 | | 12±2 | | n.s. |
| **Comorbidities** | | | | | | | |
| **Smoke history** | 50 | 50 | 50 | | - | | n.s. |
| **Hypertension (%)** | 42 | 43 | 42 | | - | | n.s. |
| **Dyslipidaemia (%)** | 62 | 71 | 58 | | - | | n.s. |
| **Diabetes (%)** | 35 | 100 | 0 | | - | | <0.0001 |

^a^ *p*<0.05 *vs*. IHD, Diabetic

^b^ *p*<0.05 *vs*. IHD, Non-Diabetic

N.S. Not significant

**Supplementary Table II - Immunofluorescence antibodies used on cardiac tissues**

| **Antigen** | **Antibody (species)** | **Supplier** | | **Cat #** | | **Antigen retrieval** | | **Dilution** | **Incubation time and temperature** | **Secondary Antibody (fluorochrome and dilution)** | **Incubation time and temperature** |
| --- | --- | --- | --- | --- | --- | --- | --- | --- | --- | --- | --- |
| ***Human samples*** | | | | | | | | | | | |
| **CD34** | Mouse monoclonal | | Dako (Santa Clara, CA, USA) | | GA632 | | Citric buffer (pH6), 98°C, 40’ | 1:10 | 2h, 37°C | Alexa633 1:800 | 1h, 37°C |
| **BPIFB4** | Rabbit polyclonal | | GeneTex (Irvine, CA, USA) | | GTX51455 | | Citric buffer (pH6), 98°C, 40’ | 1:300 | O/N, 4°C | Alexa555 1:800 | 1h, 37°C |
| **α−Sarcomeric Actin** | Mouse monoclonal | | Sigma | | A2172 | | Citric buffer (pH6), 98°C, 40’ | 1:400 | 1h, 37°C | Cy5 1:400 | 1h, 37°C |
| ***Mouse samples*** | | | | | | | | | | | |
| **Ki67** | Rabbit polyclonal | | Abcam (Cambridge, United Kingdom) | | ab15580 | | Citric buffer (pH6), 98°C, 30’ | 1:500 | O/N, 4°C | Alexa555  1:800 | 1h, 37°C |
| **α-Sarcomeric Actin** | Mouse monoclonal | | Sigma | | A2172 | | Citric buffer (pH6), 98°C, 40’ | 1:400  1:100 | 1h, 37°C  1h, RT | Cy5 1:400  Alexa568 1:200 | 1h, 37°C  1h, RT |
| **α-Sarcomeric Actinin (cardiac)** | Mouse monoclonal | | Sigma | | A7811 | | Citric buffer (pH6), 98°C, 40’ | 1:50 | 2h, 37°C | Alexa555 1:800 | 1h, 37°C |
| **Ki67** | Rat monoclonal | | Thermo Fisher Scientific | | 14-5698-82 | | Citric buffer (pH6), 98°C, 40’ | 1:100 | 2h, 37°C | Alexa633 1:800 | 1h, 37°C |
| **BPIFB4** | Rabbit polyclonal | | GeneTex | | GTX51455 | | Citric buffer (pH6), microwave 3 x 3 min 800 W | 1:100 | O/N, 4°C | Alexa488 1:200 | 1h, RT |
| **Isolectin GS-IB4 Biotin** | - | | Life Technologies | | I21414 | | Citric buffer (pH6), microwave 3 x 3 min 800 W | 1:200 | O/N, 4°C | Streptavidin Alexa 488  1:200 | 1h, RT |
| **α-Smooth Muscle Actin - Cy3** | Mouse monoclonal | | Sigma | | C6198 | | Citric buffer (pH6), microwave 3 x 3 min 800 W | 1:400 | O/N, 4°C | - | - |
| **GFP** | Rabbit monoclonal | | Thermo Fisher Scientific | | G10362 | | - | 1:100 | O/N, 4°C | Alexa568 1:200 | 1h, RT |
| **SDF1** | Rabbit polyclonal | | Abcam | | ab9797 | | Citric buffer (pH6), 98°C, 30’ | 1:200 | O/N, 4°C | Alexa647 1:200 | 1h, RT |
| **Histone H3** | Mouse monoclonal | | Abcam | | ab14955 | | Citric buffer (pH6), 98°C, 15’ | 1:100 | O/N, 4°C | Alexa488 1:200 | 1h, RT |
| **α-MyHC** | Rabbit polyclonal | | Abcam | | ab224046 | | Citric buffer (pH6), 98°C, 30’ | 1:100 | O/N, 4°C | Alexa647 1:200 | 1h, RT |

All secondary antibodies were purchased from ThermoFisher, UK.

**Supplementary Table III: List of western blotting antibodies**

| Target protein | Supplier | Catalogue number | Dilution |
| --- | --- | --- | --- |
| BPIFB4 | GeneTex, rabbit pAb | GTX51455 | 1:1000 |
| P-eNOS Ser1177 | Cell Signaling Technology  Rabbit mAb | 9570 | 1:1000 |
| eNOS | Cell Signaling Technology  rabbit pAb | 9572 | 1:800 |
| β-Actin | Sigma-Aldrich, mouse mAb | A5441 | 1:10000 |
| β-Tubulin | Cell Signaling Technology, mouse mAb | 86298/3700 | 1:1000/3000 |
| SDF1 | Abcam, rabbit pAb | ab9797 | 1:1500 |
| P-Erk1/2 Thr202/Tyr204 | Cell Signaling Technology, rabbit mAb | 4370 | 1:2000 |
| Erk1/2 | Cell Signaling Technology, rabbit mAb | 4695 | 1:1000 |
| ANP | Abcam, rabbit mAb | ab225844 | 1:1000 |
| MYH6 (MyHC-α) | ThermoFisher, mouse mAb | GT5612 | 1:500 |
| MYH7 (MyHC-β) | GeneTex, mouse pAb | GTX100713 | 1:1000 |

**Supplementary Table IV: List of flow-cytometry antibodies**

| Target protein | Supplier | Catalogue number | Dilution |
| --- | --- | --- | --- |
| human CD3 | Biolegend | 300327 | 5ul/test |
| human CD56 | Miltenyi Biotec | 130-114-739 | 1:50 |
| human CD16 | Miltenyi Biotec | 130-113-393 | 1:50 |
| human CD19 | Miltenyi Biotec | 130-114-173 | 1:50 |
| human CD66 | Miltenyi Biotec | 130-106-421 | 1:11 |
| human CD14 | Biolegend | 325622 | 5ul/test |
| human SDF-1 | Novus Biological | IC350A | 10ul/test |

**Supplementary Table V: Study 1. Echocardiography indices in non-diabetic mice given a single systemic injection of *LAV-BPIFB4* or vehicle**

|  | vehicle | *LAV-BPIFB4* | *p* value |
| --- | --- | --- | --- |
| Heart rate (bpm) | 447 ± 5 | 456 ± 6 | 0.3189 |
| End Systolic Volume (μl) | 22.2 ± 2.91 | 29.6 ± 7.95 | 0.9372 |
| End Diastolic Volume (μl) | 54.1 ± 3.55 | 63.1 ± 8.54 | 0.3520 |
| Ejection Fraction (%) | 59.4 ± 3.57 | 60.7 ± 7.16 | 0.8774 |
| Fractional Shortening (%) | 31.2 ± 2.46 | 33.0 ± 4.89 | 0.7392 |
| Cardiac Output (mL/min) | 14.2 ± 0.97 | 16.2 ± 1.27 | 0.2461 |
| LV wall thickness (μm) | 0.805 ± 0.097 | 0.872 ± 0.050 | 0.5526 |
| E/A index | 1.286 ± 0.027 | 1.434 ± 0.049 | **0.0235** |

### Echocardiography assessment was performed 4 weeks after intravenous injection of *LAV-BPIFB4* or vehicle. Values are mean ± standard error. n = 6 per group.

**Supplementary Table VI: Echocardiography results of first part of Study 2 assessing *LAV-BPIFB4 vs. WT-BPIFB4* gene therapy in diabetic mice**

|  | | **ND (n = 8)** | | **Diabetic (n =24)** | | |
| --- | --- | --- | --- | --- | --- | --- |
| **Treatment** | |  | | **V (n = 8)** | **WT (n = 8)** | **LAV (n = 8)** |
| **Parameter** | **Age (weeks)** | **Mean ± SEM** | **t-test: ND *vs.* Diabetic V** | **Mean ± SEM** | | |
| **HR (beats/min)** | **8** | 453 ± 5 | **p = 0.04** | 431 ± 7 | 439 ± 6 | 430 ± 8 |
|  | **13** | 464 ± 5 | p = 0.17 | 448 ± 8 | 449 ± 4 | 446 ± 4 |
| **E/A** | **8** | 1.51 ± 0.04 | p = 0.12 | 1.37 ± 0.06 | 1.42 ± 0.05 | 1.41 ± 0.03 |
|  | **13** | 1.57 ± 0.06 | **p = 0.009** | 1.35 ± 0.05 | 1.35 ± 0.05 | 1.48 ± 0.04 |
| **MV deceleration (mm/sec^2^)** | **8** | -38852 ± 3506 | P = 0.32 | -33560 ± 3398 | -41169 ± 3792 | -39516 ± 2557 |
|  | **13** | -38316 ± 3849 | **p = 0.036** | -28860 ± 1173 | -30474 ± 1525 | -35640± 2203 *** ^#^** |
| **DT (msec)** | **8** | 21.5 ± 1.1 | p = 0.08 | 26.7 ± 2.6 | 21.1 ± 1.5 | 21.2 ± 1.2 |
|  | **13** | 22.3 ± 1.1 | **p = 0.006** | 28.7 ± 1.5 | 27.3 ± 1.3 **^†^** | 23.3 ± 10.9 *** ^#^** |
| **LVWT (μm)** | **8** | 1.01 ± 0.08 | p = 0.21 | 0.86 ± 0.05 | 0.83 ± 0.04 | 0.84 ± 0.05 |
|  | **13** | 0.96 ± 0.09 | p = 0.98 | 0.94 ± 0.04 | 1.01 ± 0.06 **^†^** | 0.98 ± 0.05 **^†^** |
| **ESV (μL)** | **8** | 12.6 ± 0.6 | p = 0.29 | 15.2 ± 1.5 | 13.7 ± 1.6 | 14.2 ± 1.1 |
|  | **13** | 12.6 ± 1.6 | **p = 0.017** | 17.8 ± 1.1 **^†^** | 18.8 ± 2.1 **^††^** | 14.2 ± 0.9 |
| **EDV (μL)** | **8** | 55.6 ± 1.8 | p = 0.20 | 62.1 ± 3.1 | 55.7 ± 3.1 | 55.3 ± 2.4 |
|  | **13** | 55.9 ± 3.1 | p = 0.31 | 61.5 ± 2.7 | 55.7 ± 4.4 | 64.4 ± 1.9 **^††^** |
| **LV Mass (mg)** | **8** | 138 ± 13 | p = 0.31 | 117 ± 9 | 111 ± 7 | 116 ± 4 |
|  | **13** | 145 ± 12 | p = 0.66 | 132 ± 9 | 133 ± 11 | 148 ± 11 **^†^** |
| **LV Mass/BSA** | **8** | 1.50 ± 0.72 | **p < 0.0001** | 0.90 ± 0.06 | 0.89 ± 0.04 | 0.84 ± 0.04 |
|  | **13** | 1.32 ± 0.06 | **p < 0.0001** | 0.74 ± 0.03 | 0.77 ± 0.06 | 0.79 ± 0.05 |
| **FS (%)** | **8** | 45.9 ± 0.7 | p = 0.25 | 43.3 ± 1.2 | 44.2 ± 1.9 | 43.6 ±1.0 |
|  | **13** | 45.4 ± 1.9 | **p = 0.004** | 39.5 ± 0.7 **^†^** | 39.3 ± 1.1 **^††^** | 44.6 ± 0.8 **** ^###^** |
| **SV (μL)** | **8** | 44.1 ± 1.6 | p = 0.60 | 46.3 ± 1.5 | 41.9 ± 1.5 | 41.7 ± 1.6 |
|  | **13** | 42.2 ± 1.9 | p = 0.83 | 43.4 ± 1.6 | 39.9 ± 1.8 | 49.1 ± 1.4 *** ^## †^****^†^** |
| **LVEF (%)** | **8** | 78.0 ± 0.7 | p = 0.24 | 74.9 ± 1.4 | 76.3 ± 2.0 | 75.4 ± 1.1 |
|  | **13** | 77.3 ± 2.1 | **p = 0.006** | 70.8 ± 0.9 **^†^** | 70.5 ± 1.5 **^††^** | 76.3 ± 0.9 **** ^##^** |
| **CO (mL/min)** | **8** | 20.1 ± 0.8 | p = 0.98 | 19.9 ± 0.7 | 18.6 ± 0.8 | 17.8 ± 0.7 |
|  | **13** | 19.9 ± 0.8 | p = 0.99 | 19.5 ± 0.7 | 17.7 ± 1.0 | 21.9 ± 0.7 *** ^##^** **^†^****^†^** |
| **Glucose uptake (I<0.D%)** |  | **ND (n= 3)** | | **V (n = 3)** | **WT (n = 3)** | **V (n = 3)** |
|  | **13** | 5.7 ± 0.8 | **p = 0.024** | 2.5 ± 0.4 | 1.5 ± 0.6 | - 1. ± 0.7 |

******p* < 0.05 and ****** *p* < 0.01 vs. diabetic given vehicle; **^#^***p* < 0.05 and **^##^***p* < 0.01 vs. diabetic given *WT-BPIFB4*; **^†^** *p* < 0.05 and **^††^** *p* < 0.01 vs. week 8 in the same treatment group

**Supplementary Table VII: Echocardiography results of second part of Study 2 assessing the effect of SDF-1 antagonism on *LAV-BPIFB4* gene therapy**

| **Treatment** | | **GFP + DMSO**  **(n = 7)** | **LAV + DMSO**  **(n =7)** | **Fold change** | **GFP + AMD**  **(n = 6)** | **LAV + AMD**  **(n = 6)** | **Fold change** |
| --- | --- | --- | --- | --- | --- | --- | --- |
| **Parameter** | **Age (weeks)** | **Mean ± SEM** | | | | | |
| **HR (beats/min)** | **13** | 445 ± 5 | 437 ± 9 | 1.00 ± 0.04 | 443 ± 3 | 440 ± 9 | 0.99± 0.02 |
| **E/A** | **13** | 1.31 ± 0.05 | 1.43 ± 0.05 | 1.09 ± 0.04 | 1.28 ± 0.06 | 1.37 ± 0.04 | 1.07 ± 0.04 |
| **MV deceleration (mm/sec^2^)** | **13** | -30545 ± 1550 | -35779 ± 1744 ***** | 1.12 ± 0.05 | -29349 ± 2110 | -32534 ± 2142 | 1.12 ± 0.07 |
| **DT time (msec)** | **13** | 26.7 ± 0.06 | 22.6 ± 1.0 ****** | 0.86 ± 0.04 | 27.0 ± 1.4 | 27.1 ± 1.2 | 1.01 ± 0.04 **^#^** |
| **ESV (μL)** | **13** | 11.4 ± 1.8 | 5.7 ± 1.3 ***** | 0.50 ± 0.11 | 10.2 ± 1.7 | 11.5 ± 1.8 | 1.13 ± 0.17 **^#^** |
| **EDV (μL)** | **13** | 42.7 ± 2.0 | 44.7 ± 5.3 | 1.05 ± 0.12 | 43.6 ± 2.3 | 43.6± 3.2 | 1.00 ± 0.07 |
| **LV Mass (mg)** | **13** | 96 ± 4 | 93 ± 4 | 0.97 ± 0.03 | 99 ± 7 | 101 ± 4 | 1.02 ± 0.04 |
| **FS (%)** | **13** | 42.3 ± 2.7 | 53.2 ± 3.4 ***** | 1.26 ± 0.08 | 45.1 ± 2.4 | 42.2 ± 2.2 | 0.94 ± 0.05 **^##^** |
| **SV (μL)** | **13** | 31.3 ± 1.1 | 36.8 ± 3.1 | 1.10 ± 0.08 | 33.4 ± 0.9 | 32.0 ± 1.8 | 0.96 ± 0.05 |
| **LVEF (%)** | **13** | 73.9 ± 3.3 | 84.1 ± 3.1 ***** | 1.14 ± 0.04 | 77.2 ± 2.7 | 74.1 ± 2.6 | 0.95 ± 0.03 **^##^** |
| **CO (mL/min)** | **13** | 13.9 ± 0.5 | 15.8 ± 0.9 ***** | 1.14 ± 0.06 | 14.7 ± 0.4 | 14.0 ± 0.6 | 0.95 ± 0.04 **^#^** |

******p* < 0.05 and ****** *p* < 0.01 vs. diabetic given GFP; **^#^***p* < 0.05 and **^##^***p* < 0.01 vs. fold change in the DMSO groups
