# Supplementary figures and images for: Transfer of a human gene variant associated with exceptional longevity improves cardiac function in obese type 2 diabetic mice through induction of the SDF‐1/CXCR4 signalling pathway
Source: Eur J Heart Fail. 2020 May 8;22(9):1568–81. doi: 10.1002/ejhf.1840 (PMC8220375; doi:10.1002/ejhf.1840)

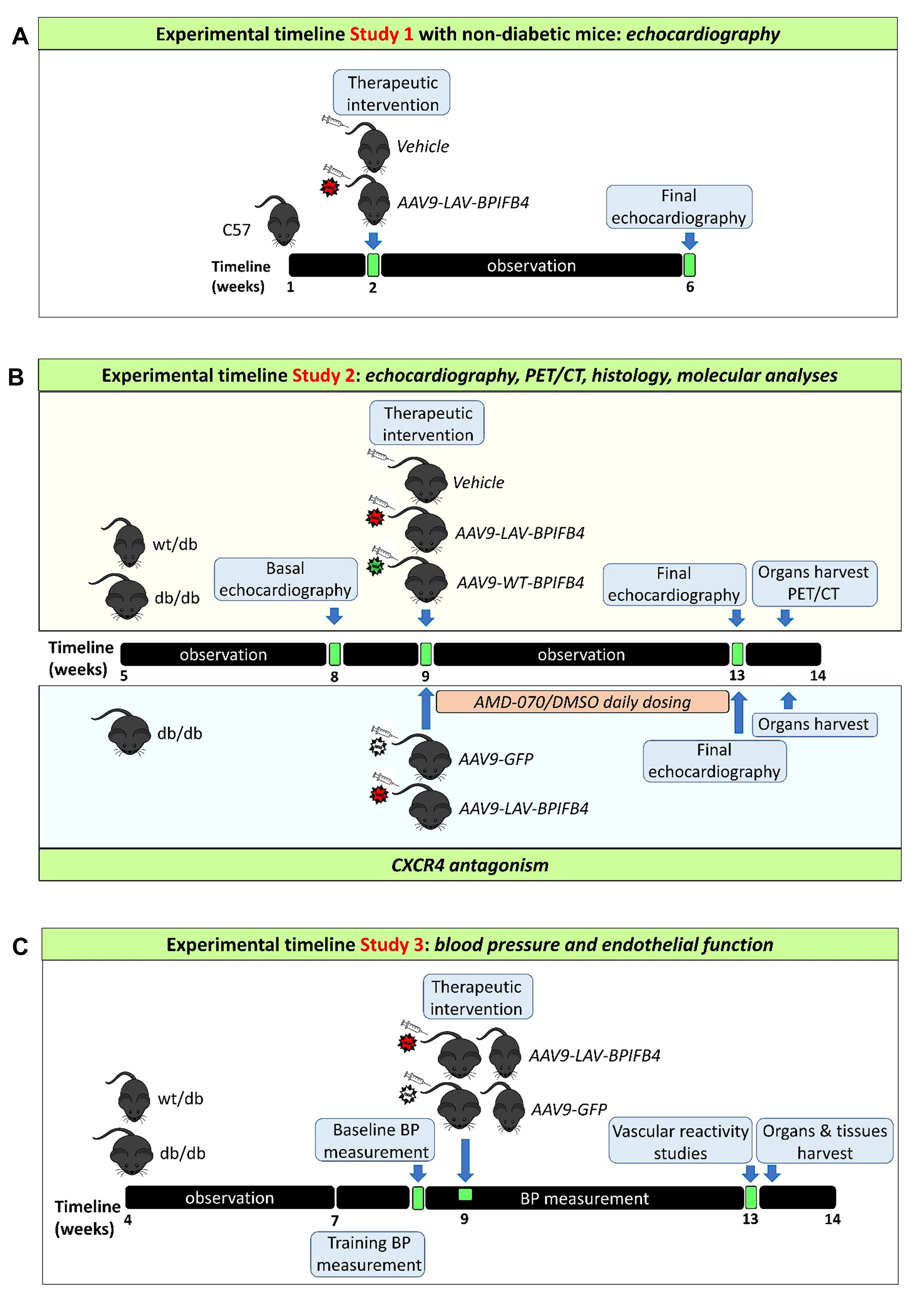

Supplement: Supplementary file 2 — Figure S1. Experimental in vivo protocols. [file EJHF-22-1568-s009.TIF]

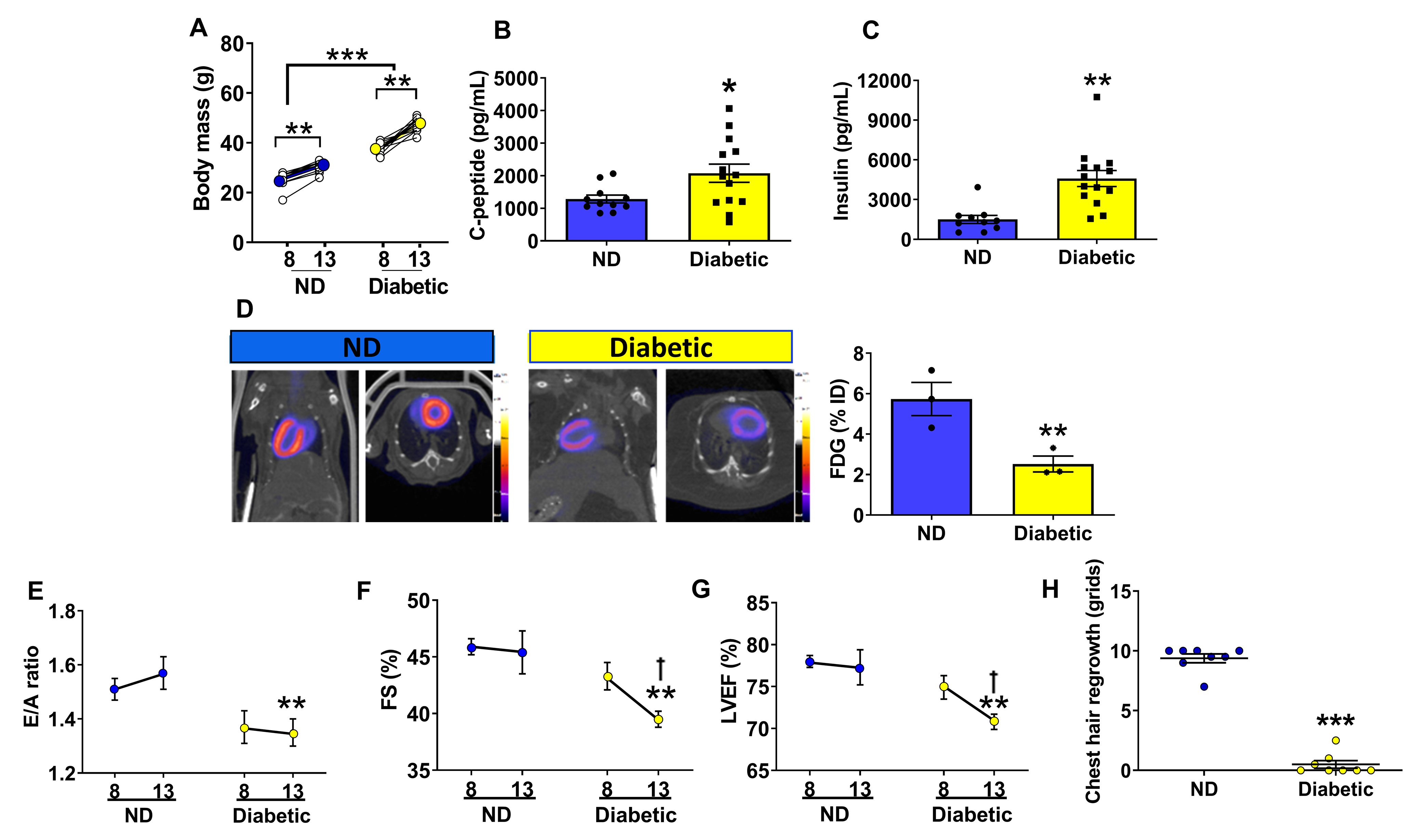

Supplement: Supplementary file 3 — Figure S2. Comparison between diabetic and non‐diabetic mice at 8 and 13 weeks of age. (A) Body weight. (B,C) Bar graphs showing peripheral blood levels of C‐peptide (B) and insulin (C). (D) FDG uptake by the heart. (E‐G) Echocardiography parameters: E/A ratio (E), fractional shortening (F) and left ventricular ejection fraction (G). (H) Chest hair regrowth. [file EJHF-22-1568-s008.TIF]

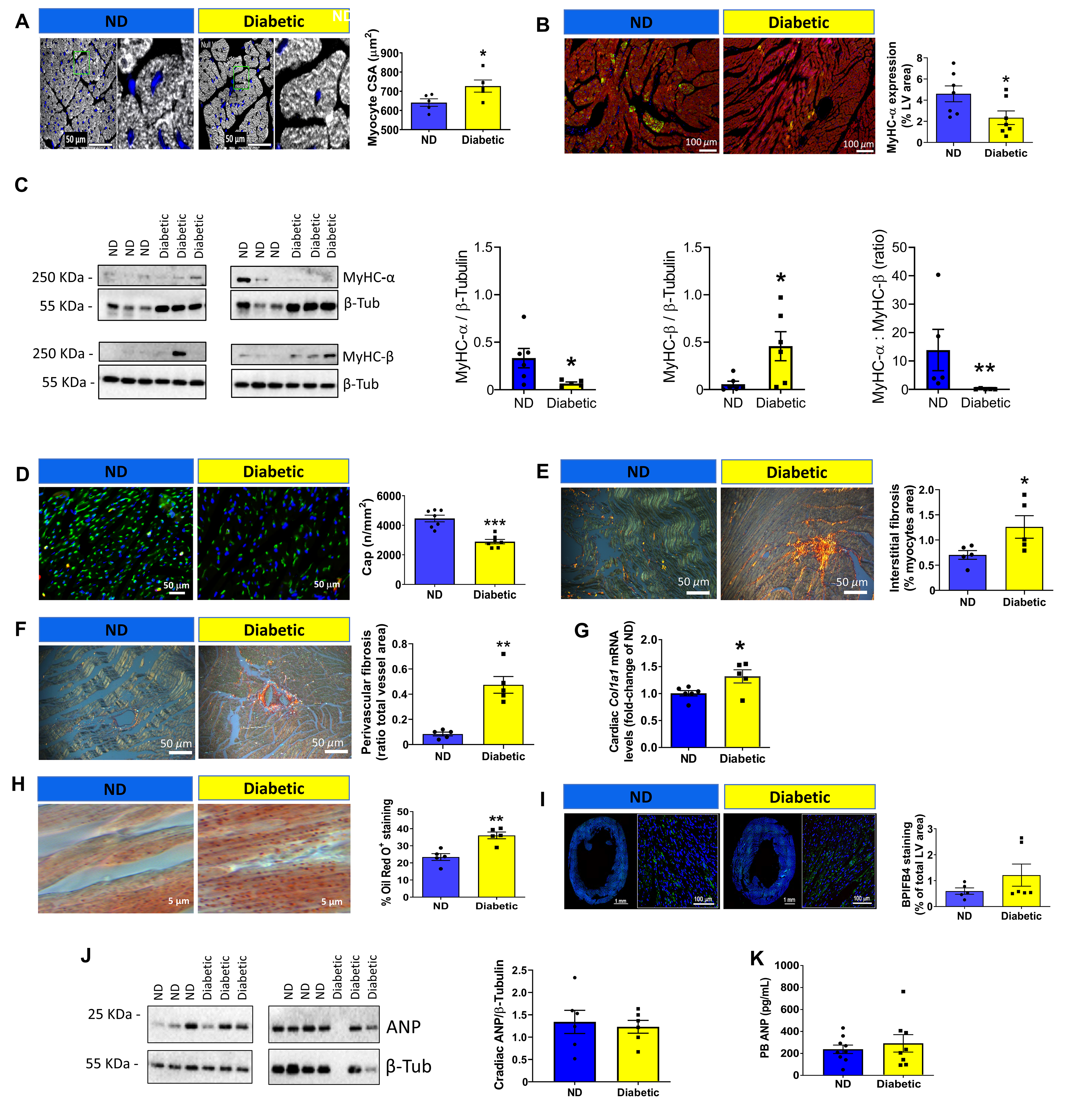

Supplement: Supplementary file 4 — Figure S3. Comparison between diabetic and non‐diabetic mice at 13 weeks of age. (A) Myocyte cross‐sectional area. (B) Expression of MyHC‐α. (C) Western blot of the cardiac MyHC isoforms alpha and beta. (D) Capillary density. (E,F) Interstitial (E) and perivascular (F) fibrosis. (G) Cardiac collagen 1A1 mRNA levels. (H) Oil red O showing lipid accumulation. (I) BPIFB4 expression. (J) Western blot of cardiac ANP. (K) Peripheral blood immunoreactive ANP levels. [file EJHF-22-1568-s010.TIF]

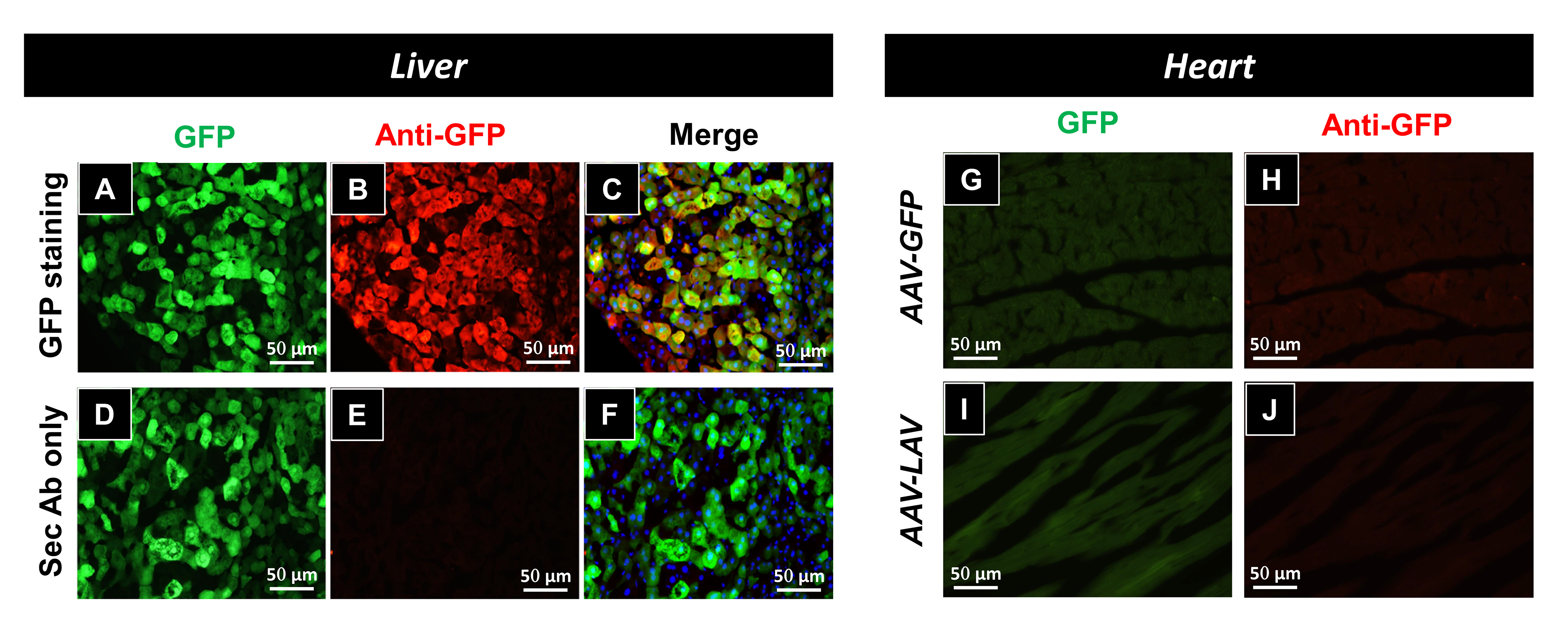

Supplement: Supplementary file 5 — Figure S4. (A–F) Representative immunohistochemistry images of livers transduced with AAV9‐GFP. An anti‐GFP antibody (B) was used to confirm the specificity of the green signal from GFP (A). Merged fluorescent signals (C). A control in which the primary antibody was omitted is shown in (D–F). (G–J) The heart of mice injected with AAV9‐GFP do not show any GFP expression (G,H). AAV9‐LAV‐BPIFB4‐transduced mice were used as controls (I,J). [file EJHF-22-1568-s001.TIF]

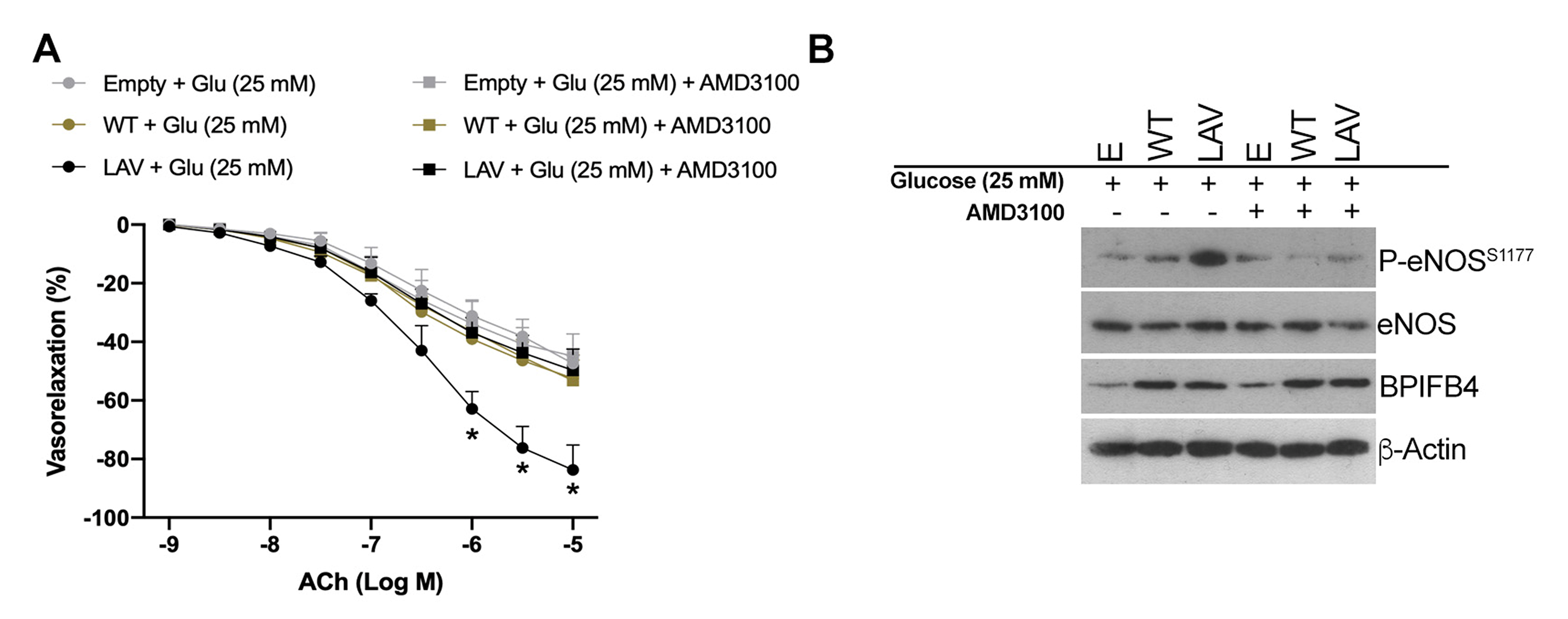

Supplement: Supplementary file 6 — Figure S5. Comparison between diabetic mice at 13 weeks of age. (A) Murine Bpifb4 mRNA expression in the hearts. (B) Plasma levels of BPIFB4. [file EJHF-22-1568-s002.TIF]

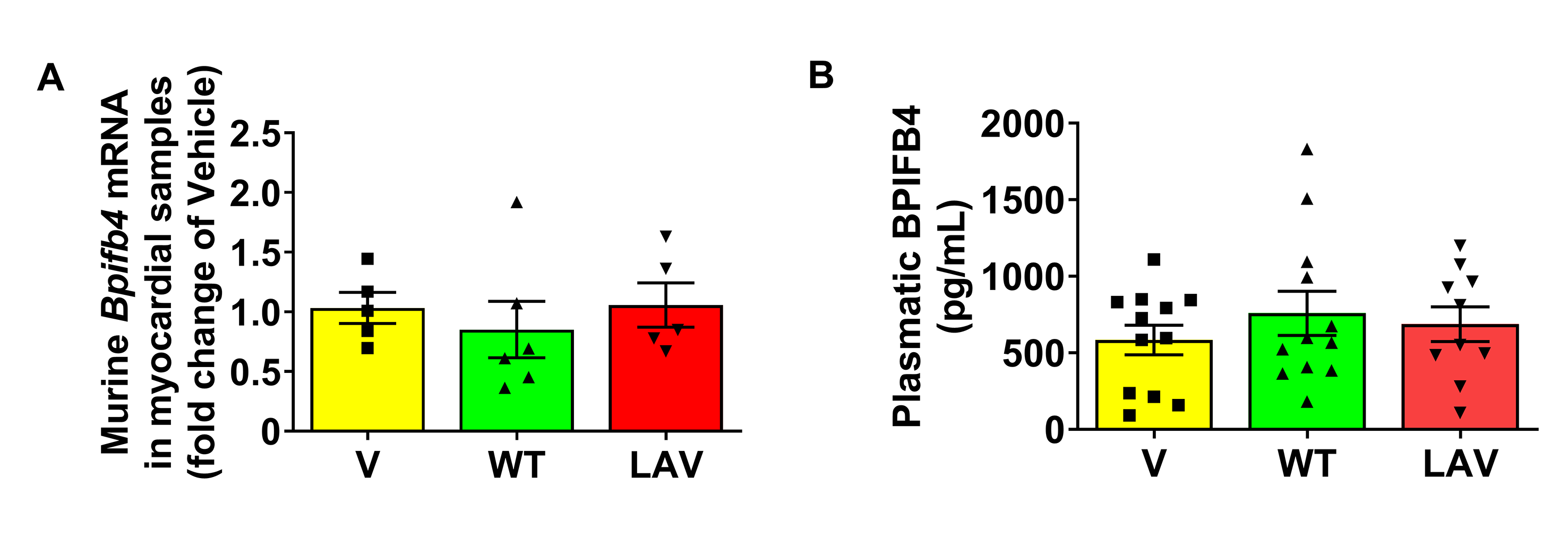

Supplement: Supplementary file 7 — Figure S6. Proliferation of cardiomyocytes. (A,B) Abundance of Ki67‐positive cardiomyocytes. (C) Phosphorylated histone H3 staining (nuclear green fluorescence) showing very few positive cells expressing the marker. [file EJHF-22-1568-s003.TIF]

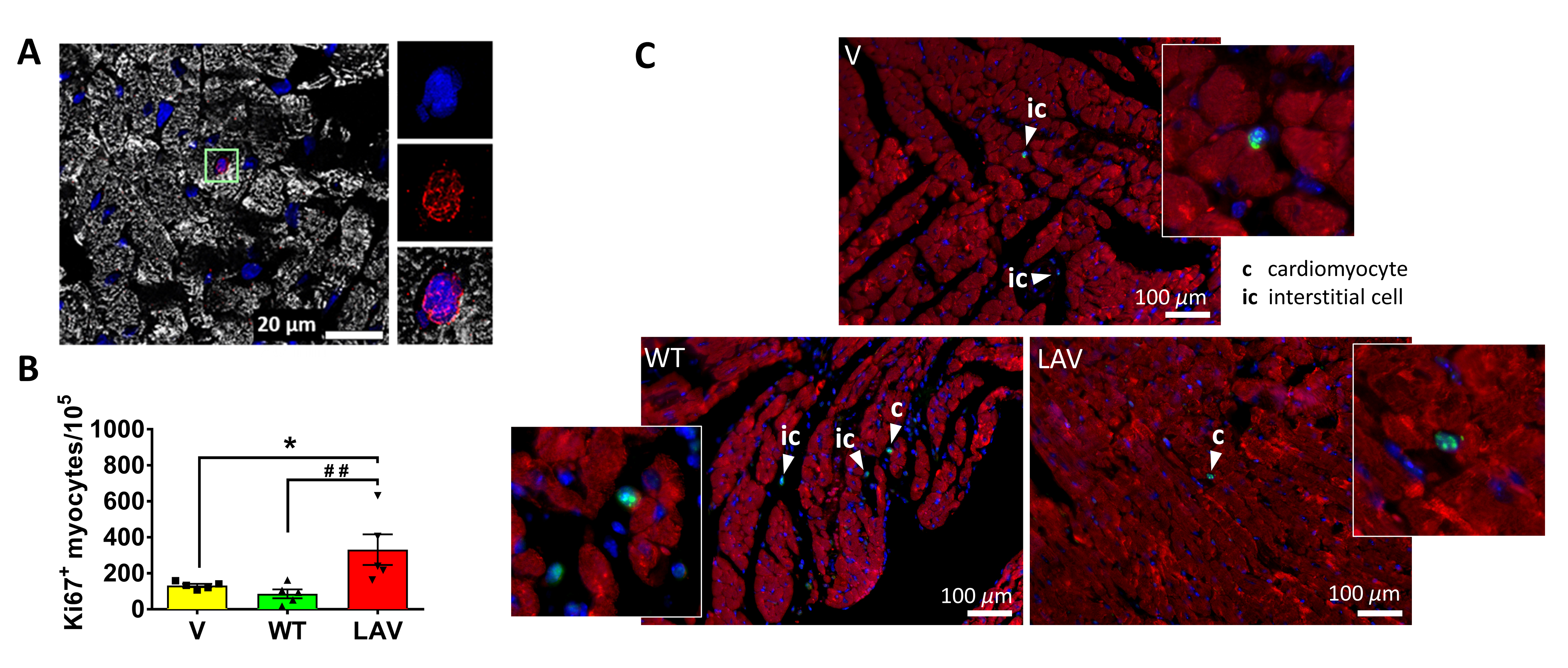

Supplement: Supplementary file 8 — Figure S7. Expression of BPIFB4, SDF‐1 and cardiac MyHC‐α in hearts of three diabetic mice treated with LAV‐BPIFB4. [file EJHF-22-1568-s004.TIF]

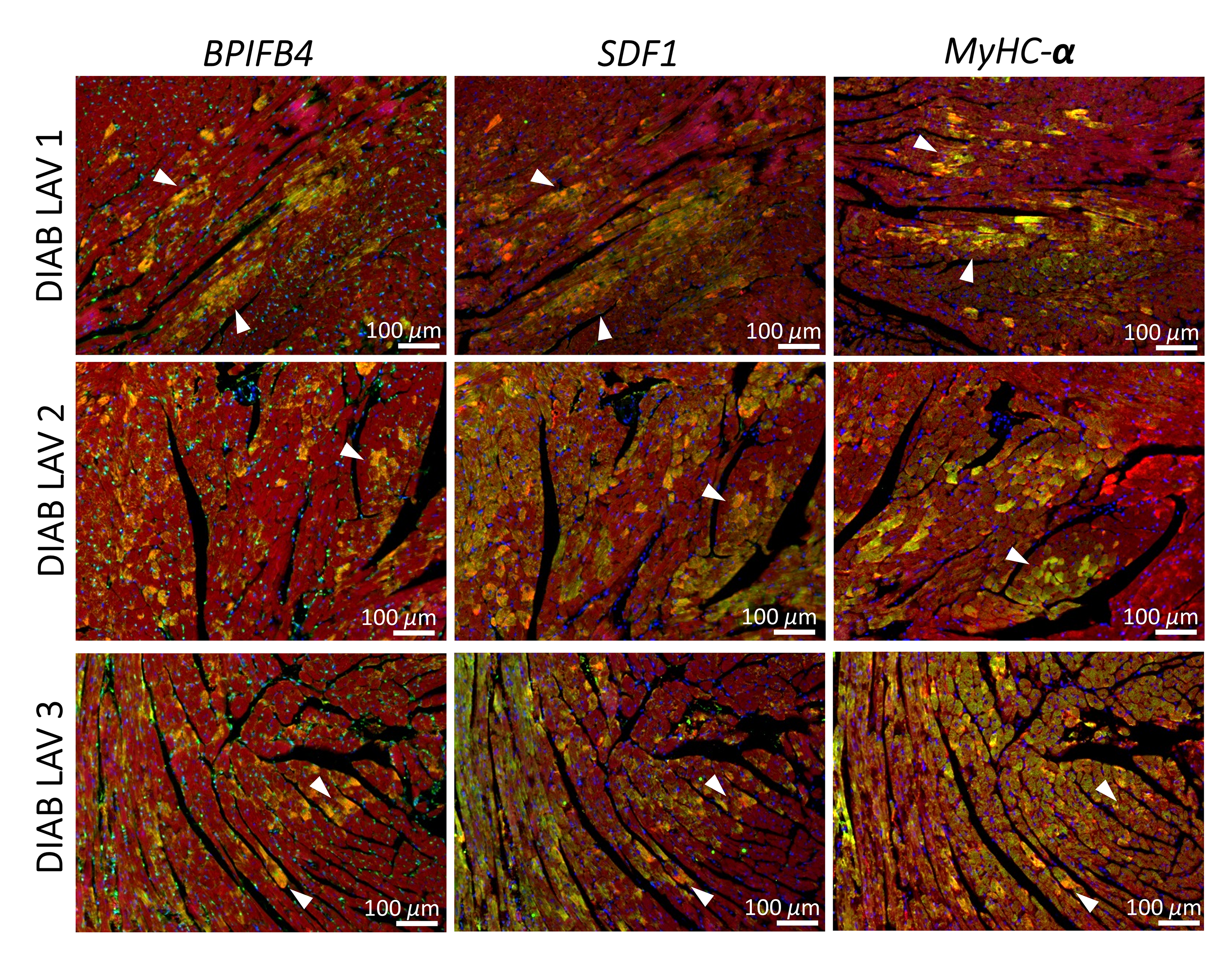

Supplement: Supplementary file 9 — Figure S8. Cell signalling activated by LAV‐BPIFB4 and antagonised by AMD‐070 in mice hearts. (A) Erk1/2 immunoblots. ( B ) Densitometry analysis. (C) Cardiac MyHC‐α and ‐β immunoblots. (D) Densitometry analysis. [file EJHF-22-1568-s011.TIF]

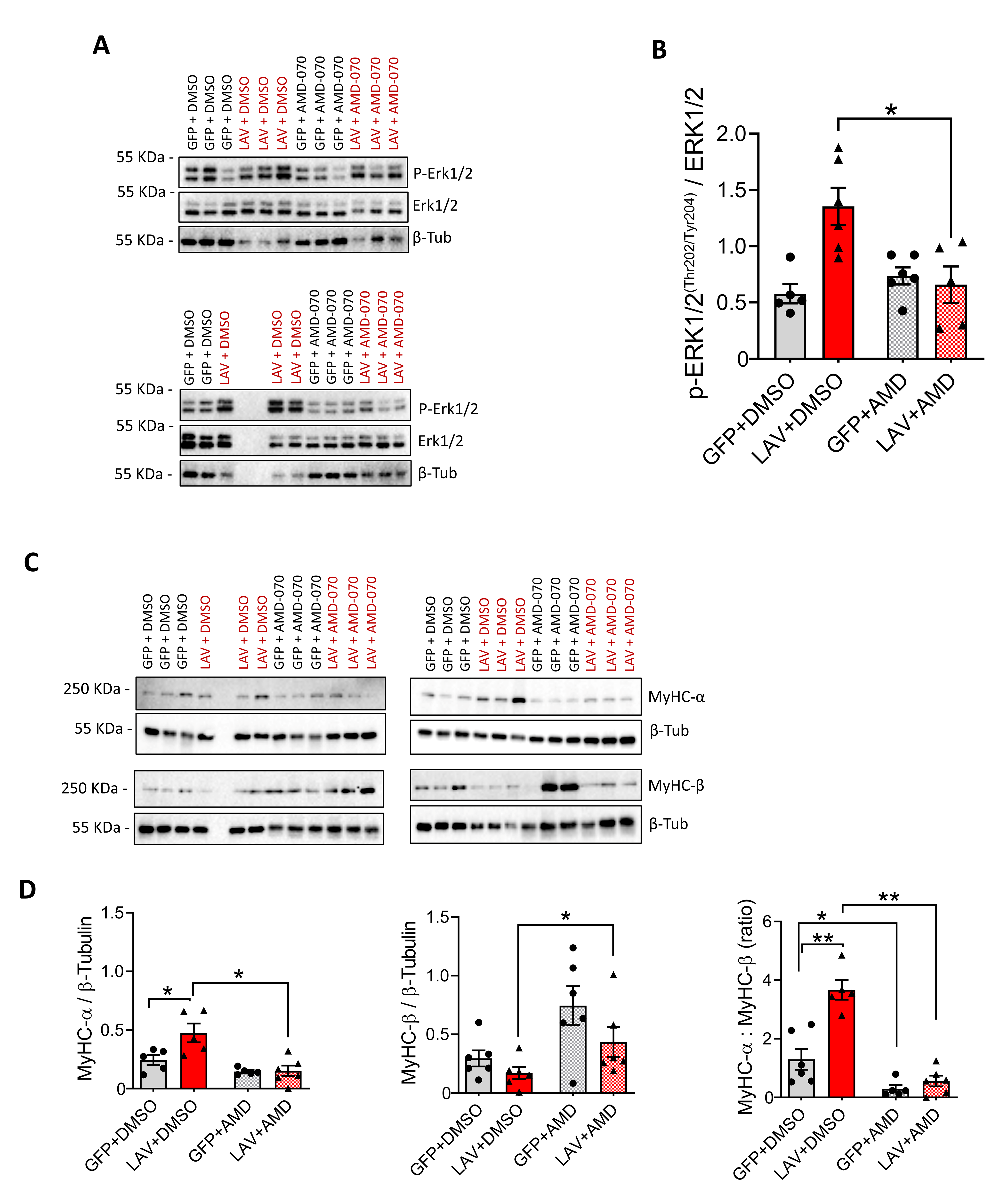

Supplement: Supplementary file 10 — Figure S9. Vessels transfected with LAV‐BPIFB4 are protected from high glucose‐induced endothelial dysfunction through a CXCR4‐mediated mechanism. (A) Graphs showing vasorelaxation induced by acetylcholine. (B) Western blot for endothelial nitric oxide synthase in the groups with or without AMD3100 treatment. [file EJHF-22-1568-s006.TIF]

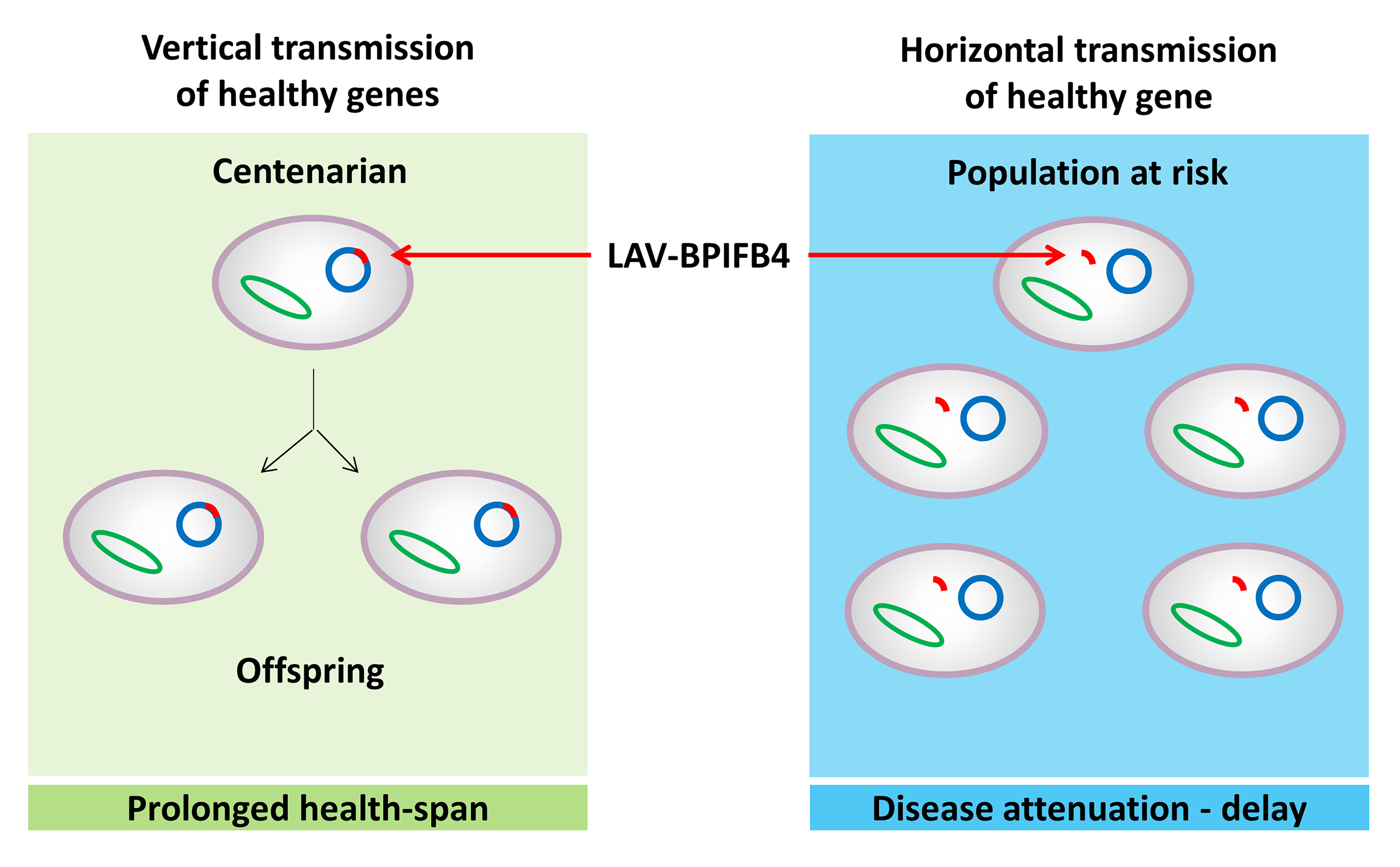

Supplement: Supplementary file 11 — Figure S10. Translational perspective. Here, we present translational evidence for a novel salutary approach inspired by the successful case of centenarians, who escape major age‐related disease and vertically transmit genetic protection against disease to offspring. Our study demonstrates that horizontal transfer of a human longevity‐associated gene variant alleviates diabetic cardiomyopathy. The encoded protein could become a novel therapeutic product to extend health‐span in cardiovascular patients. [file EJHF-22-1568-s007.TIF]
